# Protein import motor complex reacts to mitochondrial misfolding by reducing protein import and activating mitophagy

**DOI:** 10.1038/s41467-022-32564-x

**Published:** 2022-09-02

**Authors:** Jonas Benjamin Michaelis, Melinda Elaine Brunstein, Süleyman Bozkurt, Ludovico Alves, Martin Wegner, Manuel Kaulich, Christian Pohl, Christian Münch

**Affiliations:** 1 Institute of Biochemistry II, Goethe University Frankfurt am Main, Faculty of Medicine, Theodor-Stern-Kai 7, Building 75, 60590 Frankfurt, Germany; 2 Buchmann Institute for Molecular Life Sciences, Frankfurt am Main, Germany; 3 Frankfurt Cancer Institute, Frankfurt am Main, Germany; 4 Cardio-Pulmonary Institute, Frankfurt am Main, Germany; 5 Present Address: Discovery Neuroscience, AbbVie Deutschland GmbH & Co KG, Knollstrasse 50, 67061 Ludwigshafen, Germany

**Keywords:** Protein translocation, Mitochondria, Mitophagy, Protein aggregation

## Abstract

Mitophagy is essential to maintain mitochondrial function and prevent diseases. It activates upon mitochondria depolarization, which causes PINK1 stabilization on the mitochondrial outer membrane. Strikingly, a number of conditions, including mitochondrial protein misfolding, can induce mitophagy without a loss in membrane potential. The underlying molecular details remain unclear. Here, we report that a loss of mitochondrial protein import, mediated by the pre-sequence translocase-associated motor complex PAM, is sufficient to induce mitophagy in polarized mitochondria. A genome-wide CRISPR/Cas9 screen for mitophagy inducers identifies components of the PAM complex. Protein import defects are able to induce mitophagy without a need for depolarization. Upon mitochondrial protein misfolding, PAM dissociates from the import machinery resulting in decreased protein import and mitophagy induction. Our findings extend the current mitophagy model to explain mitophagy induction upon conditions that do not affect membrane polarization, such as mitochondrial protein misfolding.

## Abstract (web summary)

Mitophagy activation is mediated by mitochondrial depolarization. Here, the authors show that mitochondrial protein misfolding can activate mitophagy in a depolarization-independent manner mediated by a protein import reduction.

## Introduction

Mitochondrial protein import is essential for mitochondrial biogenesis and function. Nearly all mitochondrial proteins are encoded in the nucleus and thus synthesized as precursors in the cytosol^1^. Mitochondrial matrix and inner mitochondrial membrane (IMM) proteins are synthesized with an N-terminal mitochondrial targeting sequence (MTS) that is recognized by outer membrane receptors^2^. In a membrane potential (∆Ψ)-driven manner, they are channeled from the translocase of the outer membrane (TOM complex) to TIMM50 and the translocase of the inner membrane (TIM) complex^3–5^. Matrix-targeted proteins are actively pulled through the TIM translocon by the ATP-driven pre-sequence translocase-associated import motor (PAM) complex. The PAM machinery interacts with the TIM complex for protein import and is composed of PAM16 (also called TIMM16), TIMM44, mitochondrial heat shock protein 70 (mtHSP70, also called HSPA9) and its co-chaperones GrpEL1/2, and DnaJC19 (also known as TIMM14). HSPA9 binds to stretches of newly imported proteins to pull them through the import pore^6,7^. The mitochondrial-processing peptidase (PMPCA and PMPCB) cleaves off the pre-sequence to allow the mature proteins to fold into their native structure^8^.

According to the prevailing mitophagy model, the membrane potential breaks down during mitochondrial dysfunction and serves as trigger for PINK1/PARKIN-dependent mitophagy, which ultimately removes damaged mitochondria^9,10^. PINK1 functions as key mediator for mitophagy induction. In healthy mitochondria, PINK1 is partially imported, cleaved by processing peptidase and PARL and then retrogradely translocated to the cytosol and degraded by the proteasome^8,11,12^. In damaged mitochondria with a loss in membrane potential, PINK1 cannot be imported through the IMM and is instead stabilized at the TOM complex and the outer mitochondrial membrane (OMM). Here, the PINK1 kinase accumulates, recruits and activates the E3 ubiquitin ligase PARKIN, leading to poly-ubiquitylation of OMM proteins and ultimately degradation of dysfunctional mitochondria by mitophagy^9,10^. Mutations in PINK1 and PARKIN have been identified in early-onset Parkinson’s disease (PD) patients and confirmed in in vivo models^13,14^. Disruption of the mitochondrial protein import machinery and subsequent mitochondrial dysfunction and induction of mitophagy play key roles for neuronal health and are found to be impaired in PD^15,16^. Understanding the molecular mechanisms resulting in mitophagy induction is essential for interpreting and modulating the processes underlying neurodegeneration.

Strikingly, a number of conditions, including mitochondrial protein misfolding, have been described to induce PINK1/PARKIN-dependent mitophagy without mitochondrial depolarization^17–20^. Consequently, there ought to be additional mechanisms that can lead to mitophagy. Understanding these mechanisms will be important to understand depolarization-independent mitophagy events observed during proteostasis perturbation and, e.g., sperm development^17,19^.

Here, we identified loss or inactivation of the PAM complex as a potent mitophagy inducer, mediated by modulating mitochondrial protein import. A number of genetic and pharmacological stress conditions caused decreased matrix-targeted protein import, resulting in mitophagy induction without the need of a collapsed mitochondrial membrane potential. Under proteostasis stress conditions, the PAM complex became insoluble, sequestered from TIM and prevented active protein import. We show that reduced mitochondrial protein import was sufficient to induce mitophagy and that this process was mediated by the PAM complex upon accumulation of misfolded mitochondrial proteins.

## Results

### Loss of mitochondrial protein import motor components induces mitophagy in human cells and *C. elegans*

**Fig. 1:**
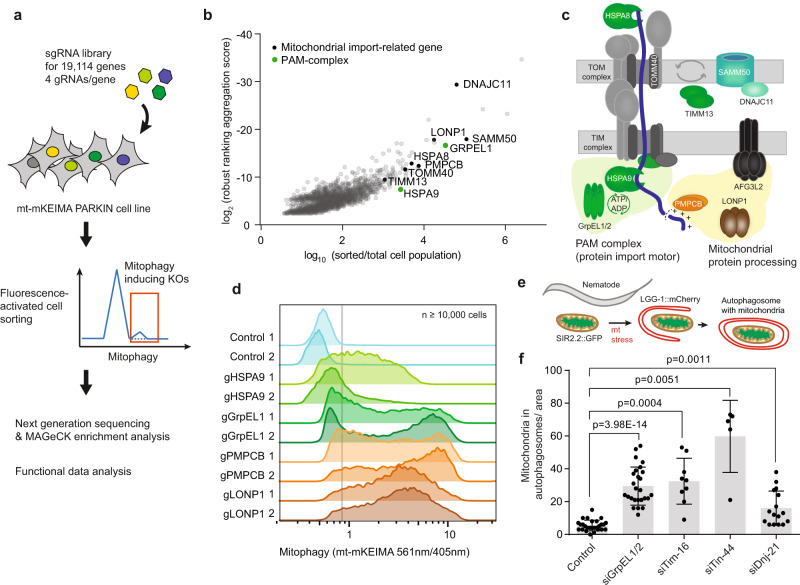
Loss of mitochondrial protein import motor components induces mitophagy in human cells and *C. elegans*. **a** Experimental scheme of a genome-wide CRISPR/Cas9 screen to identify genes that induce mitophagy when knocked-out. HeLa FlpIn cells expressing the mitophagy reporter *mt-mKEIMA* and *PRKN* (synonym PARKIN) were infected with a lentiviral particle library. Cells exhibiting induced mitophagy (high mt-mKEIMA 561 nm/405 nm ratio) were sorted and analyzed by next-generation sequencing. **b** Scatter plot presenting MAGeCK algorithm-based enrichment of targeted genes and determined robust ranking aggregation value of this gene in positive selection (sorted versus total population). Data shown in Supplementary Data 1. **c** Depiction of the mitochondrial import machinery and mitochondrial protein processing. Gene depletions identified in b as mitophagy inducers were labeled. **d** Validation of mitophagy-inducing gene knock-outs from b that are part of the matrix import machinery. Indicated genes were knocked-out individually by two gRNAs and mitophagy induction monitored by flow cytometry analysis of mt-mKEIMA. The gray line indicates the upper value of negative controls. **e** Experimental scheme to assess mitophagy in *C. elegans* using the autophagosomal marker LGG-1::mCherry (red) and mitochondrial matrix marker SIR2.2::GFP (green). **f** Quantification of co-localization of green mitochondria with red LGG-1 upon RNAi knock-down of components of the PAM complex in *C. elegans*. Bar graphs indicate median values ±s.d. (*n* = 27 control worms compared to *n* = 26 grpel1/2, *n* = 9 tim-16, *n* = 5 tim-44 and *n* = 16 dnj-21 worms, p-values were calculated by two-sided unpaired *t* test). See also Supplementary Fig. 1. mt mitochondrial.

We aimed at identifying additional pathways capable of inducing mitophagy that may explain polarization-independent mitophagy. Thus, we carried out a genome-wide genetic screen to identify genes that are crucial for mitochondrial function and activate mitophagy when depleted. HeLa cells expressing PARKIN were transduced with Cas9 and four guide RNAs per gene for 19,144 genes in total^21^ and mitophagy flux was monitored by flow cytometry using mitochondrial matrix-targeted (mt)-mKEIMA (Fig. 1a). mt-mKEIMA shifts its excitation maximum in the low-pH environment of autolysosomes, allowing ratiometric analyses of mitophagy flux^22^. Cells exhibiting increased mitophagy were sorted and knocked-out genes were revealed by next-generation sequencing. We identified 68 targeted genes in the sorted cell population that were significantly enriched over 1000-fold when compared to the unsorted population (Fig. 1b, Supplementary Fig. 1a and Data 1). Enriched genes included mitochondrial proteostasis genes, such as cytosolic and mitochondrial *HSP70, LONP1, AFG3L2*, and *PMPCB*. Strikingly, this group of genes prominently contained components of the PAM complex and mitochondrial protein import in general (Fig. 1b, c, Supplementary Fig. 1b). To validate the findings of the screen and the capacity of PAM complex components HSPA9 and GrpEL1 to induce mitophagy when depleted, we monitored mitophagy flux after individual knock-outs using two guide RNAs per gene obtained from the screen. Consistent with the genome-wide screen, deletions of HSPA9 (mitochondrial HSP70) or its nucleotide exchange factor GrpEL1 significantly induced mitophagy (Fig. 1d, Supplementary Fig. 1c, d). To determine whether the importance of the PAM components for preventing mitophagy is a conserved feature, we monitored changes in mitophagy flux upon RNAi-mediated loss of PAM complex components in *Caenorhabditis elegans*. Mitophagy was examined by analysis of mitochondrial matrix protein NAD-dependent protein deacylase (Sir2.2) and its co-localization with autophagosomal Protein LGG-1 (Fig. 1e)^23–25^. RNAi depletion of PAM components in adult worms led to co-localization of autophagosomes and mitochondria in living animals (Fig. 1f, Supplementary Fig. 1e, f), indicating that mitophagy induction upon loss of the PAM complex was conserved from *C. elegans* to human cells.

### Decreased mitochondrial protein import induces mitophagy without requiring depolarization

**Fig. 2:**
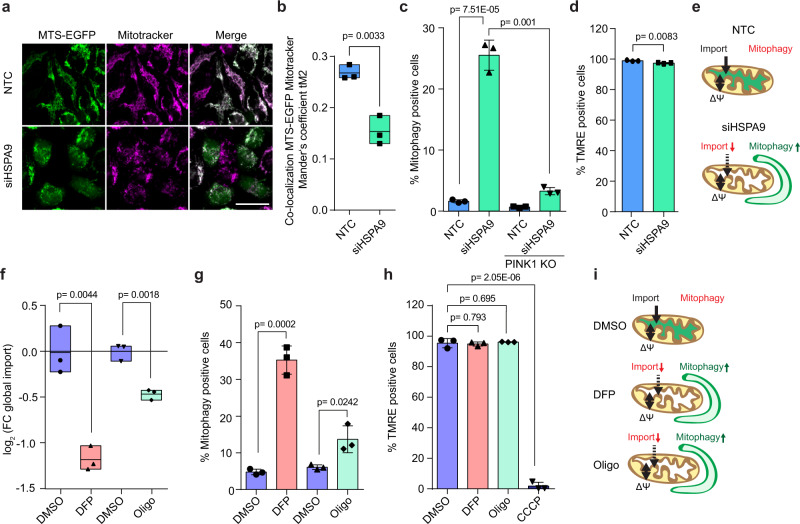
Decreased mitochondrial protein import induces mitophagy without requiring depolarization. **a** MTS-EGFP-inducible HeLa FlpIn cells (PARKIN-expressing) were treated with HSPA9 siRNA for 96 h. Doxycycline (dox) added 15 h before imaging. Mitochondrial localization of EGFP was assessed by Mitotracker Deep Red FM staining and microscopy. Scale bar 25 µm. **b** Co-localization image analysis of a. 100 EGFP-positive cells examined over *n* = 3 biological replicates. Minimum-maximum values and mean as central line shown. **c** HeLa FlpIn TRex mt-mKEIMA wild type (WT) or PINK1 KO cells (PARKIN-expressing) were treated with HSPA9 siRNA for 96 h. Cells showing increased 561 nm/405 nm mt-mKEIMA ratios were considered mitophagy-positive. Mean ±s.d. for *n* = 3 shown. **d** Mitochondrial membrane potential measurement of HeLa FlpIn TRex PARKIN cells with HSPA9 siRNA for 96 h, dox for last 15 h. Cells were stained with TMRE and categorized by gating in TMRE-positive, as untreated or TMRE-negative as depolarized, CCCP treated cells. >5000 fluorescent cells per sample analyzed. Mean ±s.d. for *n* = 3 shown. **e** Schematic overview depicting the effects of HSPA9 knock-down on mitochondrial import, membrane potential and mitophagy induction (from **a**–**d**). **f** Monitoring mitochondrial protein import by pulsed-SILAC proteomics of extracted mitochondria after treatment with 10 µM oligomycin (oligo) or 1 mM deferiprone (DFP) compared to DMSO. Cell line as d. SILAC pulse for last 2 h applied. SILAC labeled peptides included in MitoCarta 2.0 used for protein import quantification. Workflow depicted in Supplementary Fig. 2g. Minimum-maximum values, mean as central line for *n* = 3 shown. **g**, **h** Assessment of mitophagy flux by mt-mKEIMA (**g**) or membrane potential by TMRE (**h**) in cells treated with DMSO, oligomycin or DFP. As depolarization control CCCP treatment for 2 h included. Mean ±s.d. for *n* = 3 shown. **i** Schematic illustration of oligomycin and DFP effects on mitophagy, protein import and membrane potential. For all experiments, two-sided unpaired *t* test method was performed.

The PAM complex is required for matrix-targeted protein import^26^. It binds to the TIM and interacts with translocating proteins to aid in their import. We next analyzed whether the expected import defects caused by loss of PAM function can explain mitophagy induction. Knockdown of HSPA9 prevented mitochondrial protein import, indicated by the increase of the matrix marker protein MTS-EGFP^27^ outside of mitochondria (Fig. 2a, b and Supplementary Fig. 2a, b). Loss of HSPA9 led to the accumulation of full-length PINK1 (Supplementary Fig. 2c), while PINK1 knock-out cells^28^ exhibited a significantly reduced mitophagic flux, demonstrating that HSPA9 depletion triggered a PINK1-dependent mitophagy pathway (Fig. 2c). Strikingly, mitophagy induction upon import perturbation did not coincide with a loss in membrane potential (Fig. 2d). This observation suggested that defects in mitochondrial import were sufficient for mitophagy induction even without mitochondrial depolarization (Fig. 2e).

In addition to the import of matrix proteins, HSPA9 also plays an important role in folding of matrix proteins. Consequently, mitochondrial protein misfolding may contribute to the observed mitophagy induction. Thus, we tested the effect of knocking down another PAM component—PAM16. PAM16 is associated with the IMM, binds directly to TIM, and is required only for import motor function but not for HSPA9’s activity in protein folding^29,30^. PAM16 depletion reduced mitochondrial protein import and led to a significant mitophagy induction accompanied by PINK1 accumulation on mitochondria (Supplementary Fig. 2d–f). This observation supported the model that a dysfunctional PAM complex leads to induction of mitophagy due to a decrease in PAM activity and protein import.

We next aimed to further validate this hypothesis in acute induction models, by monitoring protein import, mitophagy, and membrane depolarization using compounds that are well-established to induce mitophagy without affecting protein folding^31^ and are not associated with depolarization. We employed a proteomics approach to monitor and quantify the rate of import of newly synthesized mitochondrial proteins (Supplementary Fig. 2g)^32^. Deferiprone (DFP, an iron-chelator), and oligomycin (an inhibitor of the F_1_F_0_-ATP synthase) decreased protein import (Fig. 2f, Supplementary Data 2) and induced mitophagy (increased mitophagy flux Fig. 2g and decreased mitochondrial mass Supplementary Fig. 2h, i) without a loss in mitochondrial membrane potential (Fig. 2h, i). These experiments showed for four unrelated conditions that reducing mitochondrial protein import was sufficient to induce mitophagy in a depolarization-independent manner.

### Mitochondrial protein folding stress inhibits protein import and induces mitophagy in polarized mitochondria

**Fig. 3:**
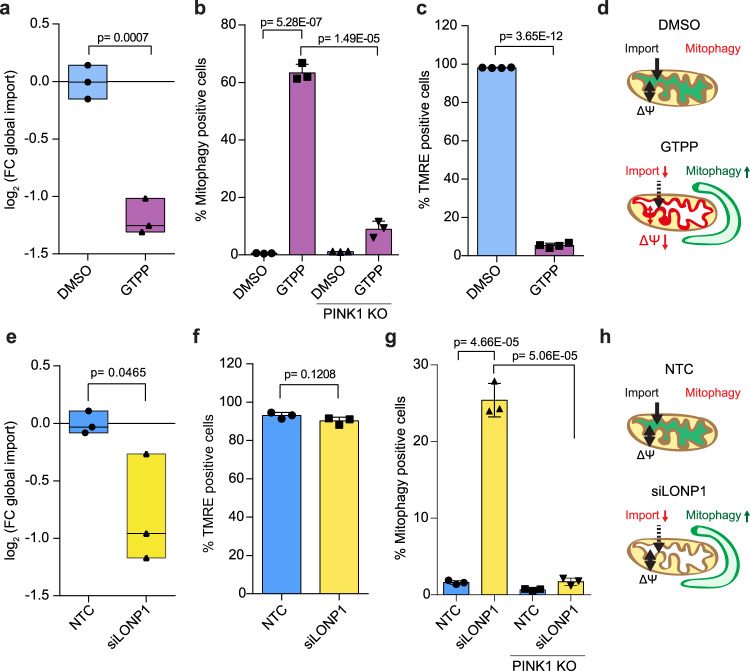
Mitochondrial protein folding stress inhibits protein import and induces mitophagy in polarized mitochondria. **a** Pulsed-SILAC mitochondrial protein import assay of HeLa FlpIn TRex PARKIN cells pre-treated 15 h with doxycycline (dox) and treated with 10 µM GTPP or DMSO for 6 h. Pulse-SILAC labeling was performed for the last 2 h. SILAC labeled peptides included in MitoCarta 2.0 were used for protein import quantification. Workflow depicted in Supplementary Fig. 2g. Minimum–maximum values and median as central line for *n* = 3 shown. **b** HeLa FlpIn TRex mt-mKEIMA (PARKIN-expressing) wild type (WT) or PINK1 KO cells treated with GTPP for 6 h. Cells showing increased 561 nm/405 nm mt-mKEIMA ratios compared to DMSO-treated cells considered mitophagy-positive. Mean ±s.d. for *n* = 3 shown. **c** HeLa FlpIn TRex (PARKIN-expressing) and stained with TMRE for mitochondrial membrane potential during GTPP treatment. Mean ±s.d. for *n* = 3 shown. **d** Schematic illustration of reduced mitochondrial import and membrane potential while mitophagy was induced by GTPP. **e** HeLa FlpIn TRex (PARKIN-expressing) cells treated for 96 h with LONP1 siRNA. Pulse-SILAC labeling carried out for last 2 h. Heavy SILAC labeled peptides included in MitoCarta 2.0 used for protein import quantification. Workflow depicted in Supplementary Fig. 2g. Minimum-maximum values and median as central line for *n* = 3 shown. **f** TMRE mitochondrial membrane potential assay for LONP1 RNAi. Mean +s.d. for *n* = 3 shown. **g** HeLa FlpIn TRex mt-mKEIMA (PARKIN-expressing) wild type (WT) or clonal PINK1 KO cells treated with LONP1 siRNA for 96 h. Cells showing increased 561 nm/405 nm mt-mKEIMA signal compared to non-targeting control-treated cells were considered mitophagy-positive. Mean ±s.d. for *n* = 3 shown. **h** Schematic illustration of reduced mitochondrial import, stable membrane potential and mitophagy induction by LONP1 RNAi. For all experiments two-sided unpaired t-test was performed to determine statistical significance.

Mitochondrial protein misfolding has been shown to induce mitophagy without a loss of membrane potential^17^ via an unknown mechanism. Based on our results that reduced protein import was sufficient to induce mitophagy (without the need of membrane depolarization), we next asked whether mitochondrial protein misfolding may activate mitophagy by this import-driven mechanism. To evaluate this hypothesis, we tested conditions directly inducing protein misfolding pharmacologically or genetically via mechanisms not involved in the import machinery: Gamitrinib-triphenylphosphonium (GTPP) inhibits the mitochondrial HSP90 (also known as TRAP1) and causes protein misfolding and induction of the mitochondrial unfolded protein response^33,34^. Treatment of cells with GTPP reduced mitochondrial protein import and induced PINK1- and PARKIN-dependent mitophagy (Fig. 3a, b and Supplementary Fig. 3a-d). However, this observation was accompanied by a loss in mitochondrial membrane potential (Fig. 3c, d).

GTPP leads to a very rapid induction of protein misfolding, which may perturb the respiratory chain acutely. Thus, we next evaluate another model for the induction of mitochondrial protein misfolding, the genetic depletion of the mitochondrial protease LONP1, which was previously shown to cause mitochondrial protein misfolding; however, requiring a longer time-span than pharmacological perturbation^35,36^. RNAi-mediated knock-down of LONP1 caused defects in mitochondrial protein import, as assessed by mitochondrial protein import assay and orthogonally by monitoring mitochondrial import of MTS-EGFP (Fig. 3e and Supplementary Fig. 3e, f). Importantly, as observed for GTPP, LONP1 depletion did not cause accumulation of mt-mKEIMA in the cytosol (Supplementary Fig. 3c, g, h). The import reduction was not accompanied by a loss in IMM membrane potential (Fig. 3f) and led to PINK1-dependent mitophagy induction, as monitored by dependency on PINK1 expression (Fig. 3g), accumulation of PINK1 on the OMM (Supplementary Fig. 3i), and PINK1 activity based on the phosphorylation of the PINK1 substrate ubiquitin (Supplementary Fig. 3i, j). The accumulation of full-length and (presumably) MTS-cleaved PINK1 on mitochondria suggests that less processing via PARL or other PINK1-proteases occurs upon treatment. Further accumulation of these PINK1 forms in PARL knock-out (KO) cells suggests the presence and activity of additional PINK1-stabilizing factors (Supplementary Figs. 2f, 3i, k, l). PINK1-independent mitophagy receptors were not found to be stabilized on mitochondria upon protein misfolding stress (Supplementary Fig. 3m).

Together and consistent with previous findings^35^, we reasoned that mitochondrial protein misfolding was able to induce PINK1/PARKIN-dependent mitophagy. We revealed that mitochondrial protein misfolding induces depolarization-independent mitophagy via the reduction of mitochondrial protein import.

### PAM complex sequestration mediates import defects upon protein misfolding to induce mitophagy

**Fig. 4:**
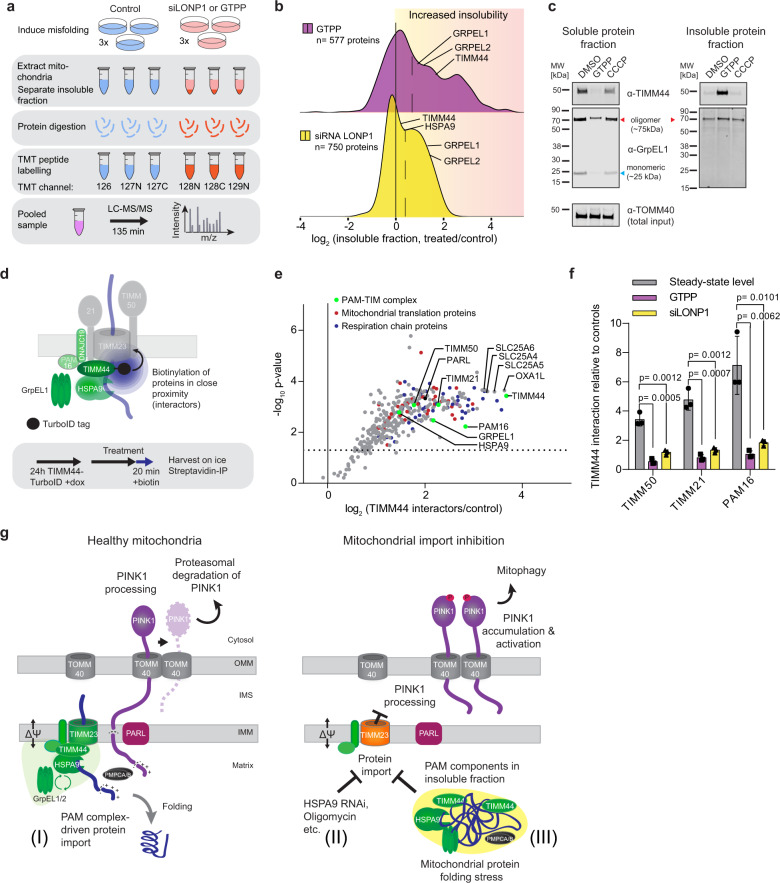
PAM complex sequestration mediates import defects upon protein misfolding to induce mitophagy. **a** Experimental scheme for the analysis of insoluble mitochondrial proteins by quantitative proteomics. **b** HeLa FlpIn TRex PARKIN cells treated for 96 h with LONP1 siRNA or with GTPP for 6 h. Log_2_ fold changes of insoluble mitochondrial protein fractions upon mitochondrial proteostasis perturbation compared to control conditions shown as density plots. Dotted line indicates the median of all identified proteins. Average of n = 3 replicates used and soluble PAM components labeled. **c** Representative immunoblots of mitochondrial soluble and insoluble protein fractions after GTPP or CCCP treatment shown for n = 3. Equal input amounts shown by TOMM40 staining of total (mitochondria). MW = molecular weight. **d** Experimental design of TurboID-TIMM44 proximity proteomics. **e** HeLa FlpIn TRex TIMM44-TurboID with 24 h dox, 20 min biotin treatment. Volcano plot presentation of proteins in close proximity of TIMM44-TurboID compared to control. Dotted line indicates significance p = 0.05 by two-sided, unpaired t-testing. **f** TIMM44-TurboID interactions with the translocon and PAM16 during protein folding stress induced by LONP1 RNAi for 96 h or GTPP treatment for 6 h, normalized to controls and interactome data from e. Data represented as mean ±s.d. for n = 3. Statistical significance determined by two-sided unpaired *t* testing. **g** Proposed model: (I) Under basal conditions, the PAM complex associates with the TIMM translocon and allows protein import. PINK1 is partially imported, leading to its degradation and prevention of mitophagy. (II) Reduced mitochondrial protein import (e.g., genetic or pharmacological perturbation) leads to PINK1 stabilization and activation on the OMM to induce mitophagy. IMM depolarization is not required for this process. (III) This mechanism is also activated during mitochondrial protein misfolding stress, in which PAM components become insoluble, lose their interaction with the translocon, and protein import is reduced. ∆Ψ = Mitochondrial membrane potential.

We next asked how mitochondrial protein misfolding may lead to protein import defects resulting in mitophagy induction. To gain insight into the fraction of proteins predominantly affected by proteostasis perturbation, we next assessed the fraction of the mitochondrial proteome becoming insoluble. We induced mitochondrial misfolding by chaperone inhibition (GTPP) or protease knock-down (LONP1 siRNA) and carried out multiplexed quantitative proteomics of the insoluble fraction (Fig. 4a). The PAM components HSPA9, GrpEL1, GrpEL2, and TIMM44 were highly sensitive to mitochondrial protein misfolding and readily accumulated in the insoluble fraction (Fig. 4b), consistent with recent findings^37^. In particular, TIMM44, the PAM component required to recruit HSPA9 and GrpEL1 to the translocon^7^, was found to significantly transition from the soluble to the insoluble fraction upon folding stress (Fig. 4b, c, Supplementary Fig. 4a–c). The effect of GTPP on misfolding was not mediated by its effect on membrane potential, as treatment with CCCP resulted in significantly less misfolding of TIMM44 and GrpEL1 compared with GTPP, despite CCCP having a stronger depolarizing effect (Fig. 4c, Supplementary Fig. 4b, c). Thus, PAM component precipitation was caused by proteostasis changes and not a general result of loss of membrane potential or import capacity. Both chemical and genetic induction of mitochondrial protein misfolding revealed similar patterns, indicating that the consequences of mitochondrial protein misfolding on the stability of mitochondrial proteins followed common principles with PAM components being vulnerable to misfolding upon stress.

To monitor changes in TIM-PAM interaction upon proteostasis perturbation and validate the PAM transition to the insoluble protein fraction, we carried out interaction proteomics of TIMM44 using proximity proteomics^38^ (Fig. 4d, Supplementary Data 4). Under basal conditions, TIMM44 interacted with both the TIM and other components of the PAM complex as expected (Fig. 4e). In addition, TIMM44 also physically connected to other mitochondrial functions, such as protein insertion into the inner membrane via interaction with OXA1L and respiration via interaction with complex I and ANT-1 (SLC25A 4,5,6), consistent with previous findings^39^ (Fig. 4e, Supplementary Data 4). Under conditions perturbing mitochondrial proteostasis, TIMM44 lost its interactions with TIM and PAM16, indicating that functional PAM complex was not located at the import machinery anymore (Fig. 4f), explaining the observed loss of protein import (Fig. 3a, e, Supplementary Figs. 3a, e, 4d). Interestingly, the analyses revealed mitophagy regulating proteins, such as AFG3L2, PARL, and PHB^11,12,40^, displaying continued interaction with TIMM44 while not accumulating in the insoluble fraction (Supplementary Data 3). This suggested a close interaction of these proteins that was not directly related to the role of TIMM44 in protein import.

Together, these results showed that mitochondrial protein folding stress caused translocation of PAM complex components from the import pore into the insoluble protein fraction, followed by mitochondrial protein import defects and mitophagy induction (Fig. 4g). These effects occurred independently of changes in the membrane potential. Thus, the identified reduced protein import during mitochondrial misfolding conditions was sufficient to induce mitophagy.

## Discussion

The current model for mitophagy induction describes a process that initiates with a loss in mitochondrial membrane potential leading to PINK1 stabilization in the OMM and ultimately autophagic degradation of mitochondria. However, mild oxidative stress or perturbation of mitochondrial proteostasis can induce mitophagy under conditions of an intact membrane potential^17,19,31,41^ (Figs. 2f–h, 3e–g). Thus, loss of membrane potential alone cannot explain mitophagy induction upon mitochondrial protein misfolding conditions. Our findings resolve this conundrum and extend the current mitophagy model by showing that reducing mitochondrial protein import by a range of conditions was sufficient to induce mitophagy without the need of a loss of mitochondrial membrane potential. Specifically, for conditions of mitochondrial protein misfolding, we identified a key role of the PAM complex. Under basal conditions, the PAM complex was soluble and located at the TIM complex to allow protein import. However, during mitochondrial protein misfolding, the soluble PAM components sequestered from the TIM complex preventing protein import and causing mitophagy induction (Fig. 4g). Proteostasis processes integrate protein synthesis (in mitochondria also import), folding, and degradation. The mitochondrial unfolded protein response (UPR^mt^) has previously been shown to reduce mitochondrial protein translation and increase folding and degradation machineries^34^. We hypothesize that the PAM complex may serve as an additional sensor of protein misfolding, responding by changing its own solubility or by binding to insoluble proteins, to control mitochondrial protein import in an attempt to restore homeostasis and to ultimately induce mitophagy. The role of PAM in the response to proteostasis perturbation might also have a longer-lived component, as we observed a decrease in HSPA9 and GrpEL1 levels that will likely cause reduced protein import into the matrix for a prolonged range of time. Thus, the PAM complex may serve short- and long-term functions in regulating import and proteostasis.

To rule out that UPR^mt^ activation leads to reduced protein synthesis and thus reduced mitochondrial protein import upon GTPP treatment, we analyzed translation rates. We observed no significant changes in translation rates for the PAM components, for TIMM23 or for TOMM40 (Supplementary Fig. 4e). However, the discovered reduced import rates upon protein folding stress would also support the mitochondrial protein quality control machinery by reducing the amount of imported proteins that require folding. Whether this would be sufficient to reverse the protein import inhibition and PINK1 stabilization needs further investigation.

The PAM components PAM16 and TIMM44 were not identified in our genome-wide screen for mitophagy inducers. However, PAM16 RNAi showed significant mitophagy induction and PINK1 stabilization on mitochondria when depleted individually. TIMM44 on the other hand has previously been shown to be essential for mitophagy induction, possibly via its interaction with ADP/ATP translocase (ANT-1)^39^. The underlying mechanism remains elusive. Our data suggests that TIMM44 carries out independent functions in humans, beside its role in the PAM complex: TIMM44 maintained interactions with key mitophagy proteins, such as PARL and ANT-1, while losing its interaction with the translocon during protein misfolding conditions.

Besides the PAM complex, our CRIPSR screen for mitophagy inducers also showed SAMM50, DNAJC11, TIMM13 and PINK1 gRNAs accumulated in the mitophagy-induced population. SAMM50 and DNAJC11 are part of the mitochondrial intermembrane space bridging complex (MIB). Activation of PINK1-PARKIN-mitophagy was previously shown for SAMM50 knock-outs^42^. Likely, DNAJC11 could trigger a similar mechanism, as it was described to be required for MIB and cristae organization^43^.TIMM13 was shown to be crucial for the insertion of TIMM23 into the IMM, which would inhibit PINK1 processing via PARL and thus could lead to mitophagy induction over time^44^. The observed identification of PINK1 in the screen was surprising at first. However, we observed elevated basal mitophagic flux in a clonal PINK1 KO cell line (Supplementary Fig. 4f, g), consistent with a compensatory induction of PINK1-independent mitophagy, as has been previously described in tissue from a PINK1^-/-^ mouse^45^.

Perturbed mitochondrial proteostasis is a hallmark of a number of neurodegenerative diseases, many of which also include defective protein import and mitophagy processes. Accordingly, loss of LONP1 has been shown to cause mitochondrial protein misfolding as part of proteinopathies, such as ALS and PD^46,47^; mutations in the import machinery (TIM and PAM complexes) are mostly lethal, but some have been described in multiple rare diseases^48–50^. However, how perturbations in these different protein quality control machineries are linked to explain similar pathologic features remained unclear. Our findings connect these different diseases mechanistically by providing PAM as a key regulatory element between folding, import and mitophagy that likely plays a role in other pathologies affecting these functions.

## Methods

### Cell culture and experimental models

Human cell lines used in this paper are human epithelial cervix-adenocarcinoma (HeLa) cells (female) and Human embryonic kidney (HEK) 293 cells. HeLa FlpIn TRex cells (gift of Christian Behrends, IBCII Frankfurt am Main, Germany, doi: 10.1016/j.molcel.2017.10.029.) were cultured at 37 °C and 5% CO_2_ in a humidified incubator in RPMI1640 medium (GIBCO 21875034) with 10% heat inactivated and sterile fetal bovine serum (FBS) (GIBCO 10270-106), supplemented with 4 µg/ml Blasticidine (Invitrogen) and 150 µg/ml Zeocin (Invitrogen). After transfection with pCDNA5 FRT/TO with an insert and co-transfection (1:9 ratio) with pOG44 Flp-recombinase expression vector (Thermo Fisher Scientific), cells were selected after 2 days for at least 14 days in 50 µg/ml Hygromycin B Gold (Invitrogen). For the mt-mKEIMA mitophagy assay, HeLa FlpIn TRex PARKIN (synonym *PRKN*) cells were transduced with pHAGE mt-mKEIMA Neo and fluorescence-activated cell sorted for mKEIMA-positive cells. HEK293 cells were cultured in Dulbecco’s modified Eagle’s medium (DMEM, Invitrogen) with 10% FBS at 37 °C and 5% CO2 in a humidified incubator. CRISPR/Cas9 knock-out cell lines were generated with lentiviral particles and transduction efficiency increased by presence of 8 µg/ml Polybrene (TR-1003, Merck Millipore), selected with 2 µg/ml Puromycin (P8833, Sigma) for 11 days. Clonal depletions were individualized in 96-well plate and verified by immunoblotting. *C. elegans* strains were maintained on standard Nematode Growth Media (NGM) as previously described^51^ and cultured at 20–25 °C.

### Constructs

MTS-EGFP was a gift from David Chan (Addgene #23214) and pCHAC-mt-mKEIMA from Richard Youle (Addgene #72342). MTS-EGFP was cloned into pLD-puro-2A-rtTA-TcVA (Addgene # 24592) by NEBuilder® HiFi DNA Assembly. mt-mKEIMA was cloned from pCHAC-mt-mKEIMA into the lentiviral over-expression vector pHAGE C-TAP and Puromycin resistance replaced by neomycin to generate stable cell lines. Two gRNA per gene from human Brunello CRISPR were used and cloned individually via BSMBI restriction into lentiCRISPRv2 (Addgene #52961).

*PRKN* was amplified from pEGFP-PARKIN WT (Addgene #45875) and cloned via Gibson cloning into pCDNA5 FRT/TO to generate doxycycline-inducible HeLa FlpIn TRex cell lines. TIMM44 cDNA was amplified from pCMV-Sport6 TIMM44 (Horizon MHS6278-202802023) and cloned in frame with GS-linker-TurboID-FLAG in a pCDNA5 FRT/TO backbone. All cloned constructs were verified by SANGER sequencing.

### siRNA knock-down

Gene knock-down was achieved by transfecting HeLa FlpIn TRex cell lines with small double-stranded interfering RNAs (siRNA). Either Dharmacon ON-TARGETplus Human SMARTPool siRNA for HSPA9, PAM16 or individual siRNA for LONP1 were compared with pooled non-targeting control (NTC) or individual NTC siRNA against firefly luciferase GL2. SiRNA was transfected with Lipofectamine RNAiMAX (Thermo Scientific, 13778150), according to manufacturer’s recommendations. Cells were cultured for 96 h after transfection until harvesting. Successful gene silencing was controlled by monitoring protein levels using immunoblots.

### Lentiviral particle production

To generate lentiviral particles, HEK293 cells were seeded to a density of 80% confluence. The standard medium was exchanged with DMEM with 1% FBS. 1/10 of culture volume Opti-MEM I (Thermo Fisher Scientific, 31985-047) was mixed with 10.5 µl Lipofectamine2000 (Thermo Fisher Scientific, 11668019) per ml medium, 1.65 µg/ml gRNA pooled library in lentiCRISPRv2 (Brunello vector) (Addgene #73178), 1.35 µg/ml pPAX1 (Addgene #12260) and 0.5 µg/ml pMD2.G (Addgene #12259). The mixture was incubated for 15 min at RT and added dropwise to the cells. The medium was exchanged again 6 h after addition.

Lentiviral particle containing supernatant was harvested 48 h after transfection and stored at −80 °C. Human Brunello CRISPR knock-out pooled library was a gift from David Root and John Doench (Addgene #73178).

Lentiviral titer was determined using HeLa FlpIn TRex cells, plated at 70% confluence. After transduction with 8 µg/ml polybrene (Sigma, H9268) and a series of 0.5, 1, 5, and 10 µl of viral supernatant, cells were incubated for 48 h at 37 °C and selected for an additional 14 days with puromycin. After selection, established colonies were counted for each dilution and the number of colonies in the highest dilution was normalized to the volume of applied virus containing supernatant to determine the lentiviral titer.

#### CRISPR mitophagy screen

Fluorescence-sorted HeLa FlpIn TRex mt-mKEIMA PARKIN cells were transduced with Human Brunello CRISPR knock-out pooled library, using 8 µg/ml polybrene and a multiplicity of infection (MOI) of 0.2. In total 4 × 10^6^ cells at 70% cell confluence were transduced yielding a coverage of 100×. Starting 2 days after transduction, cells were selected by maintaining 2 µg/ml Puromycin. Cells were collected and sorted eight days after transduction as mitophagy-positive cells or pooled as total for comparison. The mitophagy-positive gate was set to include the population of cells showing an increased 561 nm/405 nm mt-mKEIMA ratio similar to what is observed after a 6 h treatment with 10 µM antimycin A and oligomycin, while the main population of untreated cells (DMSO) or autophagy-inhibited cells (Bafilomycin A1-treated) were excluded (Supplementary Fig. 1d). The collected cells were lysed and genomic DNA extracted by GeneJet DNA purification kit (Thermo Scientific, K0721).

### Mitophagy flux mt-mKEIMA assay

Flow cytometry was performed on a BD LSRFortessa, as previously described^52^ or with BD FACSymphony A5. In brief, events were preselected for viable, single cell populations which showed KEIMA fluorescence (gating strategy shown in Supplementary Fig. 5a), dual-excitation at 405 (pH 7) and 561 (pH 4) nm with 582/15 nm emission filters for BD LSRFortessa or 610/20 nm for BD FACSymphony A5 and 610/20 nm in both cases for 561 nm excitation. The percentage of lysosomal mt-mKEIMA was calculated by analysis of the 561 nm/405 nm ratio. Data processing was done with FlowJo (v10, Tree Star). HeLa FlpIn mt-mKEIMA cells with inducible *PRKN* were pre-treated with 0.25 µg/ml doxycycline for 15 h prior to treatment or in case of siRNA treatments minimum 15 h prior to the flow cytometric measurement or microscopy. Fluorescence-activated cell sorting for individual knock-out cell lines or the genome-wide CRISPR screen were performed on a BD FACSAria™ III Cell Sorter and either collected in mixed populations or individual cells were collected in single wells of 96-well plates. Mt-mKEIMA localization in HeLa FlpIn TRex mt-mKEIMA cells with inducible *PRKN*-expression was controlled by live-cell microscopy on a Yokogawa CQ-1 with 405 nm excitation and 617/73 nm emission wavelength for neutral mt-mKEIMA and 561 nm and 617/73 nm for acidic mt-mKEIMA fluorescence. A 60x objective and 96-well plates for live-cell microscopy (Greiner 655090) were used. To determine the fluorophore localization 3-4x zoomed-in images were shown. Uncropped images are provided (see Data availability). Control experiments of HeLa FlpIn TRex (cytosolic) mKEIMA cells were performed to compare the localization with mt-mKEIMA expressing cells. HeLa FlpIn mKEIMA cells were treated for 6 h with 250 nM Torin1, Torin1 and 200 nM Bafilomycin or 10 µM GTPP. A 40x objective and 96-well plates for live-cell microscopy (Greiner 655090) were used and individual cells digitally magnified. Image recording was done for all live-cell microscopy with CQ-1 Software, image processing was performed using ImageJ 1.53c.

### Next-generation sequencing

PCR was performed with NEBNext® High-Fidelity 2× PCR Master Mix (M0541). Thermal cycler parameters were set to: initial denaturation for 5 min at 98 °C, 20 cycles of denaturation at 98 °C for 30 s, annealing for 30 s at 58 °C, extension for 40 s at 72 °C, and final extension for 5 min at 72 °C. PCR products were purified via 1% agarose gel electrophoresis and QIAquick Gel Extraction Kit (Qiagen, 28706). All samples were denatured and diluted according to the Illumina NextSeq system denature and dilute libraries guide (document # 15048776 v09, illumina.com) and sequenced on an Illumina NextSeq 500. Custom Python scripts, cutadapt 2.8 and Bowtie2 2.3.0 were used to deconvolute the raw data and determine the abundance of individual gRNAs in each sample^53,54^

### MAGeCK analysis

To identify significantly enriched/depleted gRNAs, the respective samples were analyzed with MAGeCK v0.5.6 using standard parameters and median normalization^55^. The robust ranking aggregation score provides information about significant difference between treatment and control and is in detail explained in the original publication^55^.

#### Live imaging of *C. elegans*

Appropriately staged worms in PF127, as described before^56^, were imaged using a VisiScope spinning disk confocal microscope system (Visitron Systems, Puchheim, Germany) consisting of a Leica DMi8 inverted microscope, a Yokogawa CSU X1M Dual Camscan head, and Hamamatsu sCMOS ImagEM EC- CCM cameras. Z-sectioning was performed with a Piezodriven motorized stage (Applied Scientific Instrumentation, Eugene, OR, United States). All acquisitions were performed at 20–23°C using a Leica HC PL APO ×63/1.4–0.6 oil objective. Most analysis were done in collected z-sections of 21 focal planes (1 mm apart) with 1 min intervals with a 488 and 561 nm laser at an exposure of 100 ms, for a total of 20 min.

### RNA interference in *C. elegans*

RNAi experiments were performed by feeding as previously described^57^. RNAi feeding bacteria were grown overnight (around 16-18 h) in 1 ml Luria broth with ampicillin at a concentration of 100 mg/ml and 500 ml of this culture was used to inoculate 10 ml of LB ampicillin and grown at 37°C for 6–8 h. This culture was then pelleted and resuspended in 300 ml of the same media, which was plated and kept for drying and induction on feeding plates (NGM agar containing 1 mM IPTG and 100 mg/ml ampicillin). Worms were kept on these feeding plates for 8 h, and the animals laid on these plates were analyzed 2 days later. All clones were available from the Vidal library^58^.

### Fluorescence intensities in *C. elegans* and data analysis

All quantifications of fluorescence intensities of proteins were performed on maximum intensity projection. For all measurements, background intensities were subtracted from the integrated intensity of the signals. Two-channel matching and colocalization scoring by Pearson’s correlation was used. The scatter plots represent the pixel information and were scored by Costes et al method^23^.

### MTS-EGFP mitochondrial fluorescence import assay

HeLa FlpIn TRex cells with doxycycline inducible MTS*-EGFP* and *PRKN* were treated with RNAi for 96 h, while 0.25 µg/ml doxycycline was added 24 h prior to microscopy. For GTPP treated cells, doxycycline was added only during the 6 h treatment. The cells were then stained by 50 nM Mitotracker Deep Red FM (Cell signaling 8778) for 20 min in pre-warmed RPMI 10% FBS medium. Cells were washed with PBS and incubated in RPMI 10% FBS during measurements. The Yokogawa CQ-1 with 60x magnification and automated focus was used to take live-cell images with 488 nm excitation 525/50 nm emission for EGFP and 640 nm excitation 685/40 nm emission for Mitotracker Deep Red FM. 8 images with minimum 100 cells per biological replicate in total were analyzed by JACoP ImageJ plugin^59^. The co-localization between MTS-EGFP and Mitotracker Deep Red FM was determined by thresholded M2 (tM2) Manders coefficient and gave an estimate to the amount of protein import into the matrix. The tM2 value was used as the inducible MTS-EGFP cell line contained also cells without detectable EGFP fluorescence.

### Membrane potential measurements

The mitochondrial membrane potential, the proton gradient over the inner mitochondrial membrane, was measured by tetramethylrhodamine (TMRE)^60,61^. 200 nM TMRE was used to stain cells for 30 min at 37 °C in medium. Cells were then harvested by trypsinization and EDTA, washed with cold PBS, hold on ice and subjected to flow cytometric analysis. TMRE was measured with excitation at 488 nm and a 582/15 nm emission filter. At least 10,000 cells were gated by forward and side-ward scatter for viable, single cells (Supplementary Fig. 5b), recorded and categorized by gating according to DMSO or siRNA negative and a positive control, depolarized via CCCP treatment during TMRE staining.

### Mitochondrial isolation

Cells were harvested by trypsin/EDTA treatment and washed with PBS. Cells were then resuspended in ice-cold MTE buffer pH 7.4 (270 mM D-mannitol, 10 mM TRIS, 0.1 mM EDTA) supplemented with 1× cOmplete, EDTA-free protease inhibitor cocktail (Roche, 11836170001) and lysed by sonication (25% maximum amplitude, 3× 10 s pulse, 10 s pause, Sonic Vibra Cell). For phospho-S65-ubiquitin immunoblot samples, PhosStop (Roche 4906837001) and 10 mM N-ethylmaleimide (NEM) were added to the lysis buffer. Cell debris was removed by 10 min 1400×*g* 4 °C centrifugation and the supernatant subjected to 10 min 15,000×*g* 4 °C to receive crude mitochondria, as previously described in more detail^62^. The pellet was washed once with MTE buffer and used or stored at −80 °C.

### Organelle-specific pulsed-SILAC MS sample preparation

Cells were treated for the indicated time, while the last two hours the medium was exchanged with pre-warmed heavy SILAC medium consisting of RPMI160 medium for SILAC (GIBCO 88365) supplemented with 100 μg/mL Arg10 (Cambridge Isotope Laboratories), 100 μg/mL Lys8 (Cambridge Isotope Laboratories) and 10 % FBS. Crude mitochondria were isolated and samples prepared as previously described^63^. In brief, proteins were denatured, reduced and alkylated, and then purified by methanol/chloroform precipitation. Proteins were resuspended in 8 M urea, 10 mM EPPS pH 8.2 and the protein concentration measured via Bicinchoninic Acid (BCA) Protein Assay Kit (ThermoFisher Scientific 23225). 20 µg protein were digested with 0.4 µg (1:50) LysC (Wako Chemicals) and 0.2 µg (1:100) Trypsin (Promega) 15 h at 37 °C. Peptides were purified over Empore C18 (Octadecyl) resin material (3 M Empore). 10 µg were labeled with TMT11 (Thermo Scientific, A34808), quenched and pooled for fractionation. Pierce high pH reversed phase peptide fractionation kit (Thermo Scientific 84868) was performed accordingly to manufacturer’s instructions. The fractions were dried by vacuum centrifugation for mass spectrometric measurement.

### Insoluble protein fraction sample preparation

40 µg crude mitochondria, resuspended in MTE buffer with protease inhibitor cocktail were incubated for 10 min at room temperature with 1% digitonin, if not indicated otherwise. The insoluble protein fraction was sedimented at 20,000×*g* 15 min 4 °C. The supernatant was collected, containing the soluble protein fraction and the insoluble one was resuspended in SDS-buffer, for MS in 2% SDS, 50 mM Tris-HCl pH 8, 150 mM NaCl, 10 mM TCEP, 40 mM chloroacetamide, for immunoblotting in 4× reducing SDS-sample buffer and boiled at 95 °C for 10 min.

### TurboID proximity biotinylation

HeLa FlpIn TIMM44-TurboID cells were cultured for 3 d in biotin-free medium. The TurboID-fusion gene was expressed by 0.25 µg/ml doxycycline addition 24 h prior to treatment. Proximity-labeling was induced by a 20 min incubation with 0.5 mM biotin-containing pre-warmed medium. Biotinylation was stopped by placing the cells on ice and washing 5× with ice-cold PBS. Cells were scraped off in 5 ml PBS supplemented with protease inhibitor cocktail, sedimented at 800×*g* for 3 min at 4 °C, snap frozen in liquid nitrogen and stored at −80 °C for further processing^38^.

### Streptavidin pull-down and MS sample preparation

All buffers were prepared freshly on the day of the streptavidin pull-down experiments. Frozen cell pellets were thawed on ice and incubated for 15 min in lysis buffer (8 M Urea, 100 mM sodium phosphate pH 8, 100 mM ammonium bicarbonate, 1% (w/v) SDS, 10 mM TCEP, 40 mM chloroacetamide and protease inhibitor (Sigma Aldrich)). Lysates were sonicated on ice three times for 30 s at 45% amplitude with 2 s rest between the cycles. For trichloroacetic acid (TCA) precipitation, an equal volume of 40% ice-cold TCA was added to the lysate and incubated for 1 h on ice. Precipitated proteins were spun down at 20,000×*g* at 4 °C for 10 min. Pellets were washed 3 times with 90% ice-cold acetone, air-dried and dissolved in resuspension buffer (8 M Urea, 100 mM sodium phosphate pH 8, 100 mM ammonium bicarbonate and 1% SDS) by shaking for 1 h at room temperature. After determination of the protein concentrations using the BCA Protein Assay Kit (Thermo Fisher Scientific), same protein amounts were diluted with an equal volume of mili-Q water and subject to Streptavidin pull-down. For Streptavidin pull-down 15 µl of streptavidin magnetic beads (Thermo Fisher Scientific) were prepared by washing three times with washing buffer (4 M urea, 0.5 % SDS (w/v) and 100 mM sodium phosphate pH 8). The protein lysates were mixed with streptavidin beads and gently rotated 15 h at 4 °C. The beads were washed five times using washing buffer and 10 times using washing buffer without SDS^63,64^.

For on-beads digestion, Streptavidin beads were resuspended in elution buffer (2 M urea, 200 mM EPPS pH 8.2, 8% acetonitrile) and incubated with 1 µg LysC protease per 20 µl beads for 2–3 h at 37 °C. Afterwards, the samples were dilute 1:2.5 in 200 mM EPPS pH 8.2 and digested with 0.25 µg Trypsin (Promega) 15 h at 37 °C. The supernatant was mixed with acetonitrile (final concentration 20%) and eluted peptides were labeled with TMT10. Samples were pooled and dried by vacuum centrifugation for further processing.

### Mass spectrometry

Dried peptides were resuspended with 0.5 µg/µl in 2 % (v/v) acetonitrile / 1 % (v/v) formic acid solution. Samples were shot with settings similar to previously studies^65^. First, peptides were separated on an Easy nLC 1200 (ThermoFisher Scientific) and a 22 cm long, 75 mmID fused-silica column, which had been packed in house with 1.9 mm C18 particles (ReproSil-Pur, Dr. Maisch), and kept at 45-50 °C using an integrated column oven (Sonation). Peptides were eluted by a non-linear gradient from 5%–38% acetonitrile over 120 min and subsequently sprayed into a QExactive HF mass spectrometer equipped with a nanoFlex ion source (ThermoFisher Scientific) at a spray voltage of 2.3 kV. Full scan MS spectra (350–1400 m/z) were acquired at a resolution of 120,000 at m/z 200, a maximum injection time of 100 ms and an AGC target value of 3 × 10^6^. Up to 20 most intense peptides per full scan were isolated using a 1 Th window and fragmented using higher energy collisional dissociation (normalized collision energy of 35). MS/MS spectra were acquired with a resolution of 45,000 at m/z 200, a maximum injection time of 86 ms and an AGC target value of 1 × 10^5^. Ions with charge states of 1 and >6 as well as ions with unassigned charge states were not considered for fragmentation. Dynamic exclusion was set to 20 s to minimize repeated sequencing of already acquired precursors.

### Mass spectrometry data analysis

Mass spectrometric raw data was analyzed using Proteome Discoverer 2.4 (ThermoFisher Scientific). Files were recalibrated using the Homo sapiens SwissProt database (TaxID = 9606, v. 2017-10-25) with methionine oxidation (+15.995) as dynamic modification and carbamidomethyl (Cys,+57.021464), TMT6 (N-terminal, +229.1629) and TMT6 (+229.1629) at lysines as fixed modifications, in organelle-specific pulsed-SILAC experiments, also TMT6 + K8 (K, +237.177), Arg10 (R, +10.008) were set for dynamic modifications, as described in earlier studies^65^. Spectra were selected using default settings and database searches performed using SequestHT node in Proteome Discoverer. Database searches were performed against a trypsin digested Homo sapiens SwissProt database and FASTA files of common contaminants (‘contaminants.fasta‘ provided with MaxQuant) for quality control. Fixed modifications were set as TMT6 at lysine residues, TMT6 (N-terminal) and carbamidomethyl at cysteine residues. As dynamic modifications acetylation (N-terminal) and methionine oxidation were set. After search, posterior error probabilities were calculated and PSMs filtered using Percolator using default settings. The Consensus Workflow for reporter ion quantification was performed with default settings. For the organelle-specific pulsed-SILAC experiments, peptide files were exported and heavy SILAC-labeled peptides extracted^65^. Mitochondrial proteins were annotated using the human MitoCarta 2.0^66^. Density plots were produced with R studio using ggridges and tidyverse packages^67,68^.

### Immunoblotting

Protein samples in reducing SDS-sample buffer were separated by SDS-PAGE with 4–12% or 12% Bolt Bis-Tris Plus Gels (Thermo Scientific). Proteins were transferred to 0.45 µM nitrocellulose membranes, blocked for 1 h with Intercept® (PBS) Blocking Buffer (LI-COR Biosciences, 927-70001) and incubated with a primary antibody diluted in 50% PBS 0.1% Tween, 50% Intercept (PBS) Blocking Buffer under gentle shaking for 15 h. Blots were washed three times for at least 5 min with PBS 0.1% Tween. Secondary antibodies were used as 1:15,000 in 80% PBS, 20% Intercept (PBS) Blocking Buffer and incubated for 1 h, room temperature under gentle shaking in an opaque incubation box. Blots were washed three times for at least 5 min with PBS 0.1% Tween and rinsed with PBS. Near-infrared secondary antibodies were imaged using an Odyssey CLx imager (LI-COR). Colorimetric measurement, image adjustments and quantification were done with Image Studio Lite v5.2 (LI-COR).

Antibodies used:

anti-beta-Actin (SantaCruz, sc69879, dilution 1:5000)

anti-GrpEL1 (Proteintech, 12720-1-AP, dilution 1:1000)

anti-HSP60 (Abcam, ab4679, dilution 1:10,000)

anti-HSPA9 (Abcam, JG1 clone, ab2799, dilution 1:2000)

anti-LONP1 (Proteintech,15440-1-AP, dilution 1:1000)

anti-PAM16 (Proteintech, 15321-1-AP, dilution 1:1000)

anti-phospho (S65)-Ubiquitin (Boston Biochem, A110, dilution 1:1000)

anti-PINK1 (CST, D8G3 clone, 6946, dilution 1:1000)

anti-TIMM23 (Proteintech, 11123-1-AP, dilution 1:1000)

anti-TIMM44 (Proteintech, 13859-1-AP, dilution 1:1000)

anti-TOMM20 (SantaCruz, sc17764, dilution 1:1000)

anti-TOMM40 (SantaCruz, sc 365467, dilution 1:1000)

anti-mouse-IgG-680RD (Li-Cor 926-68072, dilution 1:15,000)

anti-mouse-IgG-800CW (Li-Cor 926-32210, dilution 1:15,000)

anti-rabbit-IgG-680 (Li-Cor 926-68073, dilution 1:15,000)

anti-rabbit-IgG-800CW (Li-Cor 926-32213, dilution 1:15,000).

### Statistics and reproducibility

*C. elegans* experiments: the number of replicates per condition is mentioned for each condition or experiment individually. For each RNAi experiment, at least five biological replicates were carried out and technical replicates of these pooled. Animals and embryos with clear developmental problems or improperly mounted were excluded from our analysis. Cell culture experiments: Statistical significance for immunoblot, FACS results or global import rates were determined by two-sided unpaired or paired *t* tests as stated in the figure legends, and performed with GraphPad Prism Version 6 or Version 9 or Microsoft Excel 2016. Replication was generally done by independent biological experiments, if not stated otherwise in the figure legend. Statistical significance for the genome-wide CRISPR screen per gene were calculated via the MAGeCK algorithm as described before^55^. Reactome pathway analyses were performed with PANTHER 15.0 Fisher’s exact testing giving a measure for overrepresentation of the pathway compared to *Homo sapiens* reference list^69^. The used corrections for multiple testing are stated individually in the figure legends. No statistical methods were used to predetermine sample size. For quantified data, if not stated otherwise, mean and standard deviation (s.d.) are indicated.

### Reporting summary

Further information on research design is available in the Nature Research Reporting Summary linked to this article.

## Supplementary information

Supplementary Information

Description of Additional Supplementary Files

Supplementary Data 1

Supplementary Data 2

Supplementary Data 3

Supplementary Data 4

Reporting Summary

## Data Availability

Flow cytometric pseudocolor plots for mt-mKEIMA assays and histograms for membrane potential measurements via TMRE, as well as uncropped images of all immunoblots used for this study can be found in the Source Data file. Original full size microscopy images are accessible here 10.17632/4dc8hyjwhw.1 [https://data.mendeley.com/datasets/4dc8hyjwhw/1]. The analyzed CRISPR screen data are available in Supplementary Data 1. Next-generation sequencing raw files are available at GEO under the identifier GSE207212. The mass spectrometry proteomics raw data have been deposited to the ProteomeXchange Consortium via the PRIDE^70^ partner repository with the dataset identifier PXD022560, PXD022524, and PXD022530. The *Homo sapiens* SwissProt database (TaxID = 9606, v. 2017-10-25) was used for proteome analyses. The processed mass spectrometry datasets generated during this study and used in Figs. 2–4 and Supplementary Figs. 2–4 are available in Supplementary Data 2–4. Source data are provided with this paper.

## Source data

Source Data
