# Supplementary material for: Protein import motor complex reacts to mitochondrial misfolding by reducing protein import and activating mitophagy
Source: Nat Commun. 2022 Sep 2;13:5164. doi: 10.1038/s41467-022-32564-x (PMC9440083; doi:10.1038/s41467-022-32564-x)
Supplement: Supplementary file 1 — Supplementary Information [file 41467_2022_32564_MOESM1_ESM.pdf]

Supplementary information for the study:

Protein import motor complex reacts to mitochondrial misfolding by reducing protein import and activating mitophagy

Jonas Benjamin Michaelis[1], Melinda Elaine Brunstein[1], Süleyman Bozkurt[1], Ludovico Alves[1,4], Martin Wegner[1], Manuel Kaulich[1,2,3], Christian Pohl[1,4,°] and Christian Münch[1,2,3,*]

[1]Institute of Biochemistry II, Goethe University Frankfurt am Main, Theodor-Stern-Kai 7, Building 75, 60590 Frankfurt, Germany.

[2]Frankfurt Cancer Institute, Frankfurt am Main, Germany.

[3]Cardio-Pulmonary Institute, Frankfurt am Main, Germany.

[4]Buchmann Institute for Molecular Life Sciences, Frankfurt am Main, Germany.

°Current address: Discovery Neuroscience, AbbVie Deutschland GmbH & Co KG, Knollstrasse 50, 67061 Ludwigshafen, Germany.

*Correspondence: ch.muench@em.uni-frankfurt.de.

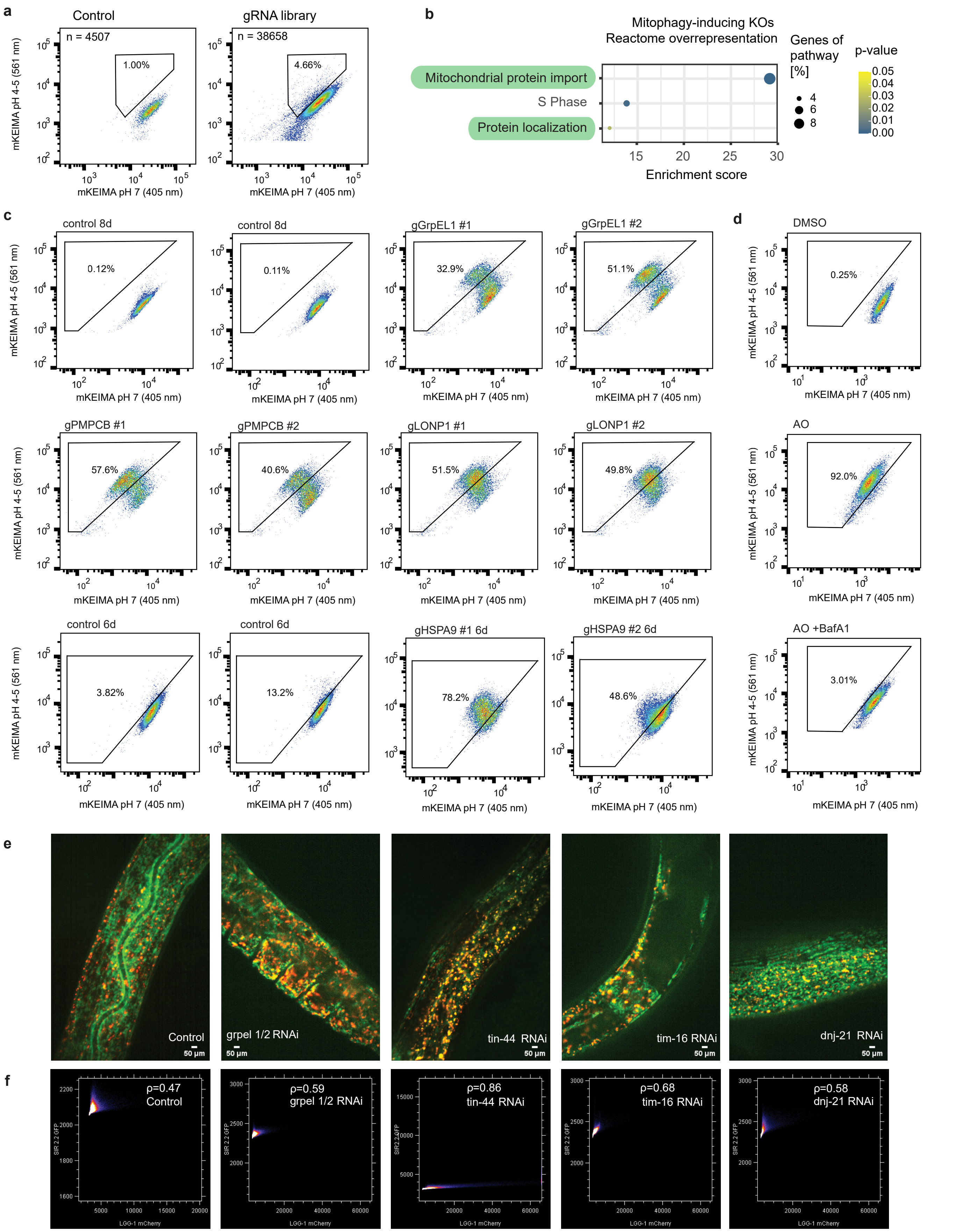

Supplementary Figure 1: Validation of candidates genes derived from the CRISPR/Cas9 screen by individual gene knock outs or knock downs in *C.elegans*. (a) Representative dot plot showing mt-mKEIMA flow cytometry analysis of CRISPR/Cas9 library-treated and untreated HeLa FlpIn TRex mt-mKEIMA PARKIN cells. Numbers of fluorescent and percentage of mitophagy-positive cells are indicated. (b) Reactome overrepresentation analysis of gene knock-outs >1000x significantly, enriched from a. Statistical significance for pathway enrichment determined by using Fishers exact testing with Bonferroni correction in PANTHER15.0. (c) mt-mKEIMA flow cytometry pseudo color dot plot showing mitophagy induction for 2 gRNA per candidate after 8 d or 6 d selection. Minimum 5,000 fluorescent cells per plot are shown. (d) Positive and negative control for mitophagy mt-mKEIMA assay showing 5 h 10 µM antimycin and oligomycin ±200 nM bafilomycin A1 compared to DMSO control. (e) *C. elegans*, expressing endogenous LGG-1::mCherry and overexpressing SIR2.2::GFP were treated with RNAi against PAM components. Representative pictures shown and done for a minimum of 5 biological replicates. (f) Two-channel matching and co-localization scoring by Pearson's correlation indicated. Scatter plot represents pixel information and was scored accordingly to the method described by Costes et al.[32].

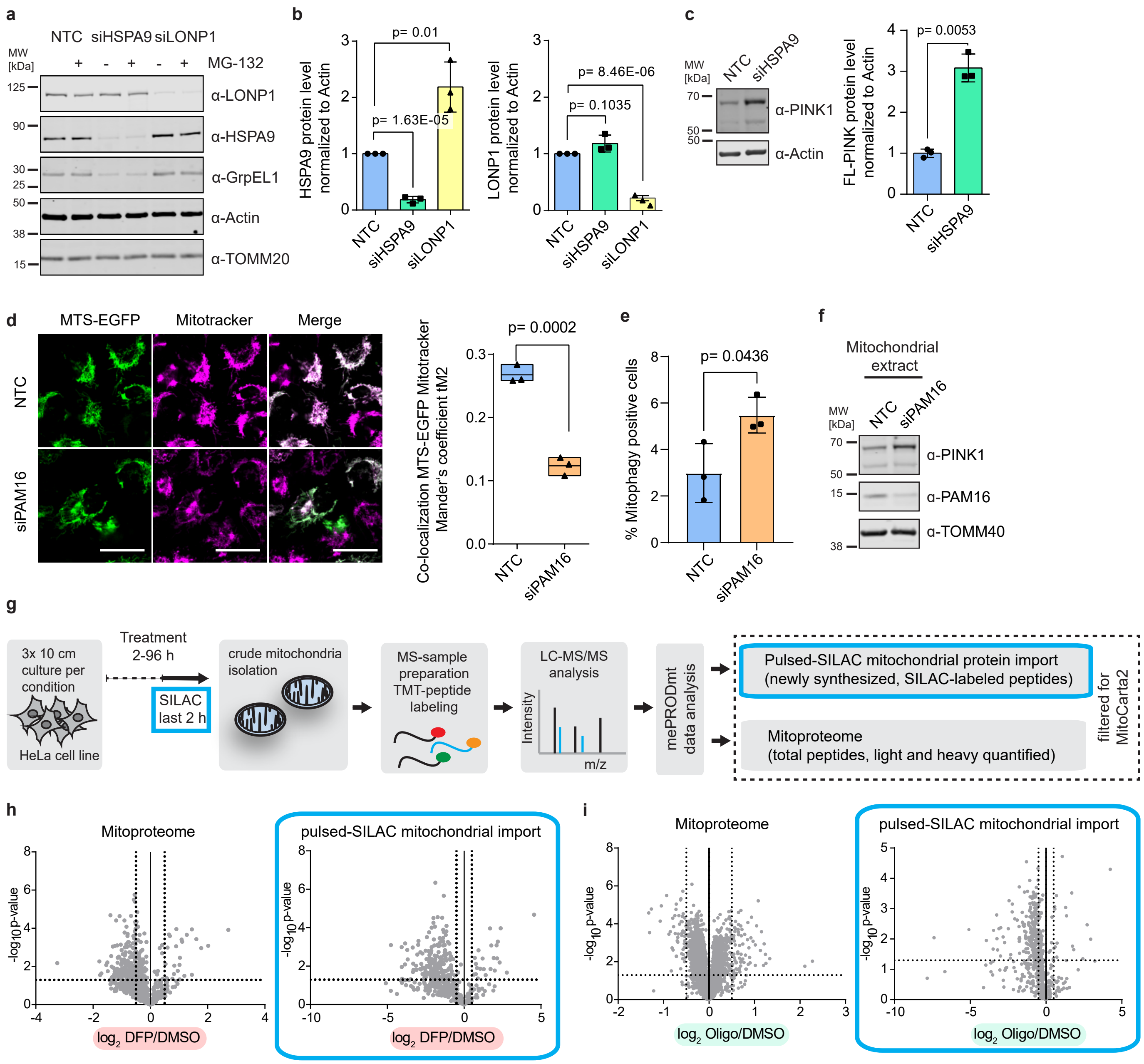

Supplementary Figure 2: Mitochondrial protein import defects induce mitophagy. (a) Representative immunoblots show protein levels after RNAi knock-down of LONP1, HSPA9, or a non-targeting control (NTC) for 96 h in HeLa FlpIn TRex PARKIN expressing cells. Samples of one experiment were loaded several times and processed in parallel. Performed for n=3. (b) HSPA9 and LONP1 protein levels of three, parallel experiments were quantified relative to Actin levels. Data shown as mean ±s.d. Significance tested by two-sided, paired t-test. (c) Representative immunoblot of HeLa FlpIn TRex PARKIN whole cell lysates stained against PINK1 after knock-down with HSPA9 or NTC siRNA for 96 h. Quantification of three, parallel experiments shown as mean ±s.d.. (d) HeLa FlpIn TRex cells with dox-inducible MTS-EGFP and PARKIN expression were treated with PAM16 siRNA for 96 h. Dox was added 15 h before microscopy. Mitochondrial localization of EGFP was analyzed via staining with Mitotracker Deep Red FM and live cell imaging (left). Scale bar 25 µm. (Right) Co-localization image analysis for n= 3 biological replicates shown as mean of 100 EGFP-positive cells per replicate, mean minimum-maximum values for replicates indicated. (e) HeLa FlpIn TRex mt-mKEIMA cells with inducible PARKIN expression were treated with PAM16 siRNA for 96 h. Dox was added 15 h before flow cytometric measurements. Cells showing increased 561 nm/405 nm mt-mKEIMA ratios when compared to main population in NTC-treated cells were considered mitophagy-positive. Mean ±s.d. for n=3. (f) Representative immunoblot (n=3) of mitochondrial extracts from 96 h PAM16 RNAi treated HeLa FlpIn TRex PINK1 IRES PARKIN dox-inducible cells. Dox was added for last 15 h. (g) Workflow illustration of pulsed-SILAC mitochondrial protein import assay measuring uptake and total abundance of mitochondrial proteins. Two data sets per sample were received, one monitoring heavy SILAC-labeled (i.e. newly synthesized) imported proteins (blue frame) and one data set quantifying all identified mitochondrial proteins to obtain the mitochondrial proteome (Mitoproteome). (h, i) Volcano plots of mitochondrial proteome and mitochondrial import (pulsed-SILAC) upon indicated mitochondrial stresses are shown for n=3. Horizontal dotted lines indicate p=0.05, vertical log2 fold change=±0.5. Significance was tested by two-sided unpaired t-test for c, d, e, h and i. MW = molecular weight.

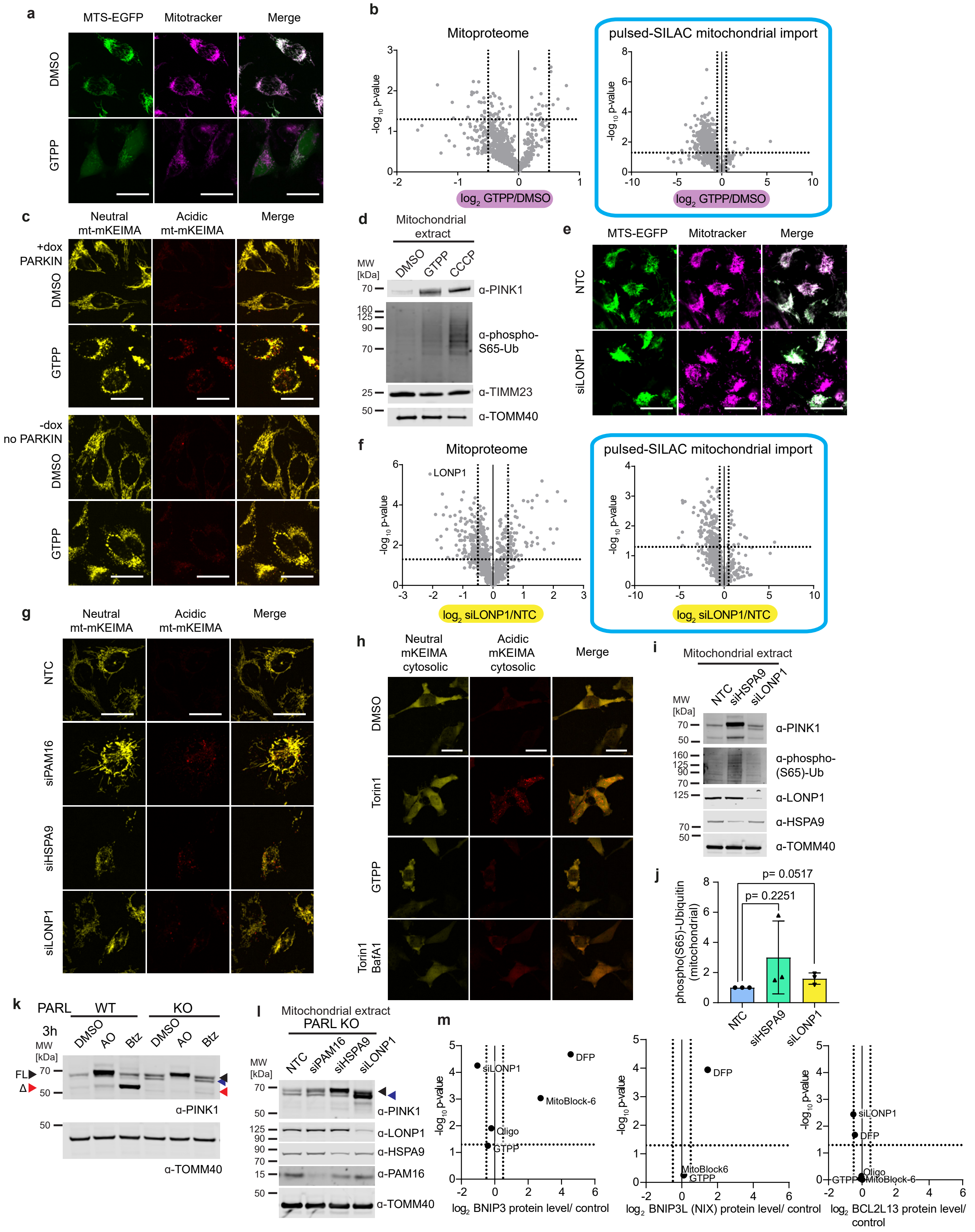

Supplementary Figure 3: Proteostasis perturbation reduces protein import and induces mitophagy. (a) MTS-EGFP-inducible HeLa FlpIn cells (PARKIN-expressing) were treated with 10 µM GTPP and doxycycline (dox) for 6 h and mitochondrial colocalization assessed by microscopy. Scale bar 25 µm. Representative images shown, n=3. (b) Mitochondrial proteome and import upon 6 h GTPP treatment shown for n=3. HeLa FlpIn PARKIN cells, pre-treated with dox for 15 h. (c) HeLa FlpIn mt-mKEIMA cells expressing PARKIN were treated with GTPP for 6 h and analyzed by live-cell microscopy. Scale bar 25 µm. (n=3). (d) Representative immunoblots (n=3) of mitochondrial extracts from HeLa FlpIn PINK1 IRES PARKIN cells with dox 15 h and treated with 10 µM GTPP or CCCP for 6 h. Samples were run on several gels, αTIMM23 loading control for αPINK1, αTOMM40 for α-phospho-(S65) ubiquitin. (e) HeLa FlpIn cells with MTS-EGFP and PARKIN expression were treated with LONP1 or non-targeting control (NTC) siRNA for 96 h. Scale bar 25 µm. n=3. (f) as b, cells were treated with LONP1 RNAi for 96 h before measurement. (g) HeLa FlpIn mt-mKEIMA cells expressing PARKIN treated with siRNA for 96 h. Scale bar 25 µm. n=3. (h) HeLa FlpIn cytosolic mKEIMA cells were treated as indicated in methods and analyzed by live-cell microscopy. Scale bar 25 µm. n=3. (i) Representative immunoblot (n=3) of mitochondrial extracts from 96 h RNAi treated, induced HeLa FlpIn PINK1 IRES PARKIN cells. (j) Quantification of phospho-(S65)-ubiquitin signal from i, in n=3. Mean ±s.d.. (k) HeLa FlpIn PINK1 IRES PARKIN cells edited by NTC or PARL gRNA treated with 100 nM Bortezomib (Btz), 10 µM antimycin and oligomycin (AO) or DMSO for 3 h to monitor PARL activity by PINK1 processing. Mitochondrial extracts analyzed via immunoblot (n=1). PINK1 shows three variants: full length (FL, ~66 kDa, black arrow head), a smaller variant (presumably processed by PMPCA/B, blue arrow head) and PARL-processed PINK (Δ, 52 kDa PINK1, red arrow head). (l) Mitochondrial extracts of HeLa FlpIn PINK1 IRES PARKIN PARL knock-out cells treated for 96 h with indicated siRNAs analyzed for PINK1 stabilization by immunoblots (n=1). (m) Mitoproteome analyses of HeLa FlpIn TRex PARKIN cells treated with DFP (24 h), oligomycin (oligo, 24 h), GTPP (6 h), MitoBlock-6 (6 h)[32] or LONP1 siRNA (siLONP1, 96 h). Three PINK1-independent mitophagy receptors were plotted for their accumulation on mitochondria upon the respective treatment. Horizontal dotted lines p=0.05, vertical log2 fold change=±0.5. Significance was tested by two-sided unpaired t-test. MW = molecular weight.

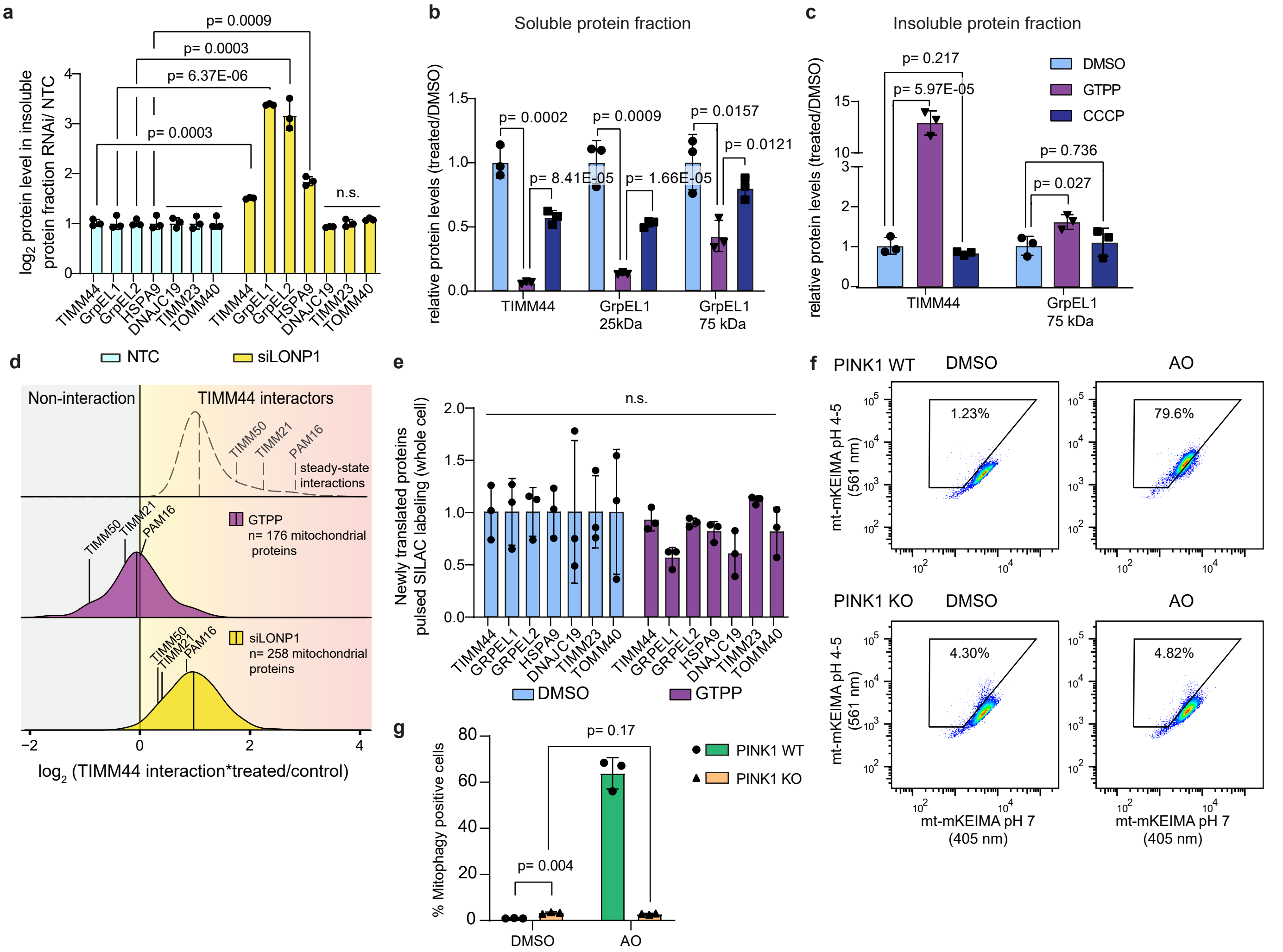

Supplementary Figure 4: TIMM44-translocon complex dissociates upon protein folding stress and PAM components accumulate in the insoluble protein fraction. (a) Bar graph representation of PAM components and TIM, TOM core proteins, shown as mean ±s.d for n=3. Significance was tested by two-sided unpaired t-test. (b, c) Quantification of TIMM44 and GRPEL1 in the soluble and insoluble protein fractions upon GTPP treatment for 6 h, in n=3 parallel experiments shown as mean ±s.d. One representative experiment was shown in Figure 4c. Significance was tested by two-sided unpaired t-test. (d) Density plot representation of TIMM44-TurboID proximity measurements upon GTPP versus DMSO (6 h) or RNAi against LONP1 versus non-targeting control (96 h) treatment shown for n=3 biological replicates. HeLa FlpIn TRex TIMM44-TurboID cells were pre-treated for 24 h with dox to express TIMM44 construct. (e) Pulsed-SILAC-based translation analysis of whole cell lysates from DMSO or GTPP treated HeLa FlpIn TRex cells. Data shown as mean ±s.d. for n=3. Significance was tested by two-sided unpaired t-test. (f) HeLa mt-mKEIMA wild type (WT) or PINK1 knock-out cells with PARKIN expression were treated with 10 µM antimycin A and oligomycin (AO) for 5 h. Dox was added 15 h before flow cytometric measurements. Cells showing increased 561 nm/405 nm mt-mKEIMA ratios compared to DMSO-treated cells were considered mitophagy-positive. (g) Three replicates of f were quantified, shown as mean ±s.d. Significance was tested by two-sided unpaired t-test.

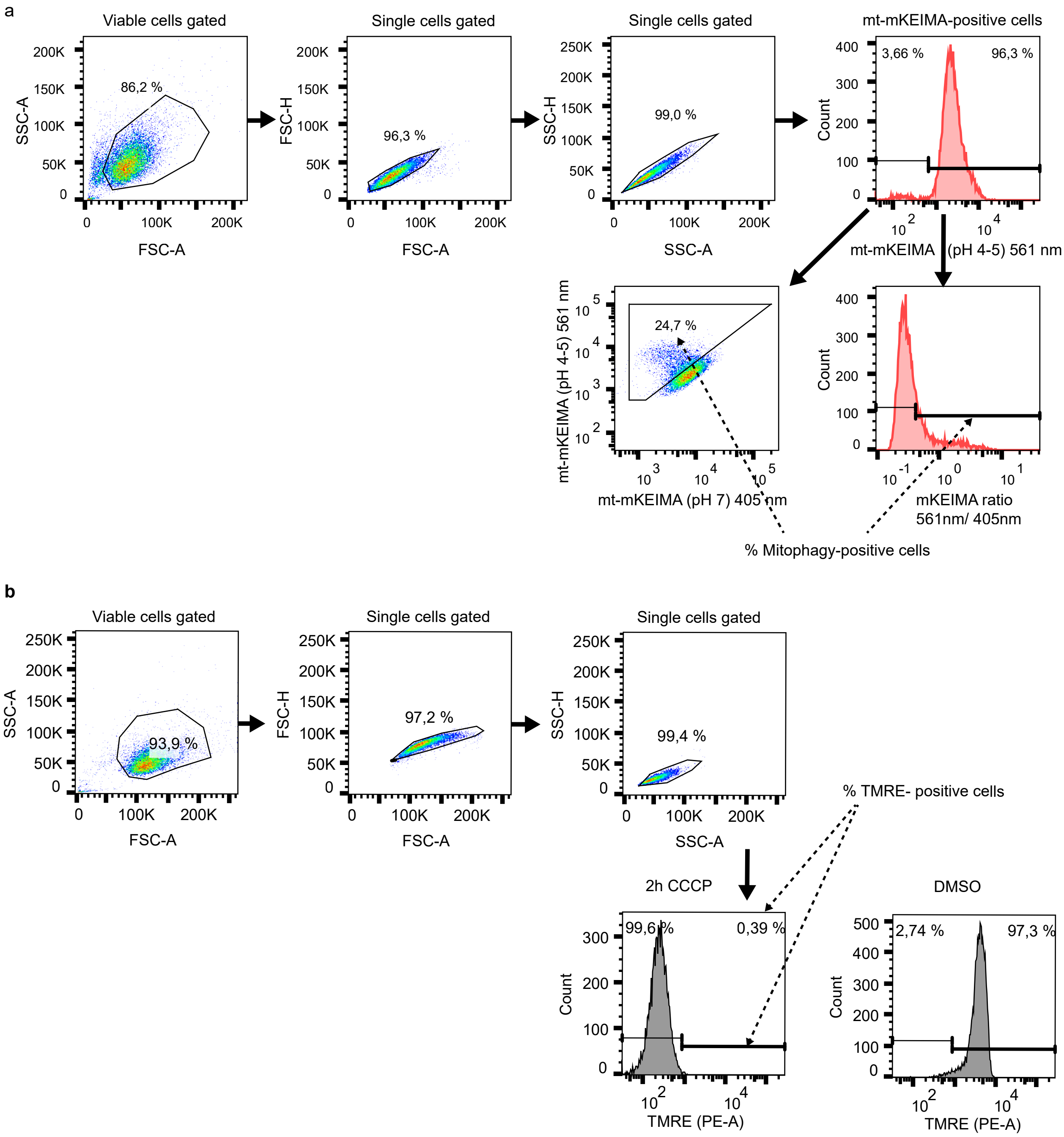

Supplementary Figure 5: Flow cytometry gating strategy. HeLa FlpIn TRex cells with dox-inducible PARKIN were used. The total event population was gated in FSC-A/SSC-A excluding debris and including viable cells, next single cells by FCS-A/H,SSC-A/H were gated and fluorescent cells either (**a**) mt-mKEIMA pH4-5 (basal level)-positive or (**b**) ratio of TMRE-positive were used for further analysis. Mitophagy analysis of mt-mKEIMA cells can be done by pseudocolor plots or histograms of the 561 nm to 405 nm channel. A minimum of 10,000 total viable cells were collected and minimum 5000 fluorescent cells for mt-mKEIMA measurements.
