# Supplementary material for: Protein import motor complex reacts to mitochondrial misfolding by reducing protein import and activating mitophagy
Source: Nat Commun. 2022 Sep 2;13:5164. doi: 10.1038/s41467-022-32564-x (PMC9440083; doi:10.1038/s41467-022-32564-x)
Supplement: Supplementary file 2 — Description of Additional Supplementary Files [file 41467_2022_32564_MOESM2_ESM.pdf]

File name: Supplementary Data 1

Description: Mitophagy CRISPR screen.

HeLa FlpIn cells expressing the mitophagy reporter mt-mKEIMA reporter were infected with a lentiviral particle library containing 77,441 gRNAs (4 gRNAs/gene for 19,114 genes). Cells exhibiting induced mitophagy were sorted and analyzed by next generation sequencing. Sorted versus total population is shown after MAGeCK v0.5.6 enrichment, represented as robust ranking aggregation score of this gene in positive selection, p-value and enrichment log10 fold change (sorted/total population). Statistical significance was calculated as described in the original publication [56]. See also Figure 1a, b.

File name: Supplementary Data 2

Description: Mitochondrial proteome and pulsed-SILAC mitochondrial protein import assay.

Mitochondrial proteome and SILAC-labelled mitochondrial fraction (protein import) log2 fold change and p-value of two-sided unpaired t-tests without adjustments for multiple testing between 24 h DFP/24 h DMSO, 24 h 10 µM Oligomycin/24 h DMSO, 6 h 10 µM GTPP/6 h DMSO and 96 h siLONP1/96 h NTC treated cells. Data is shown in Supplementary Figure 2g, h, i, 3b, f and m. MitoBlock-6 data shown in Supplementary Figure 3m was reanalyzed for PINK1-independent mitophagy receptors from a previous study [32]. Each tab contains proteome of mitochondrial extracts "(Mito-proteome)" and mitochondrial protein import data "(Import)". Tabs are labeled with its associated subfigure, SF = Supplementary Figure. Mitochondrial localization is given as Yes or No, based on MitoCarto2.0.

File name: Supplementary Data 3

Description: Quantitative proteomics of insoluble protein fraction upon misfolding stress.

Global analyses of the misfolded protein fraction upon proteostasis perturbation. Abundances of all replicates and relative fold changes between treatment and control shown. Statistical significance was determined by two-sided unpaired t-tests tests without adjustments for multiple testing. Data shown for 6 h 10 µM GTPP/6 h DMSO and 96h siLONP1/96h NTC treated HeLa cells. Data is normalized to global protein levels per channel and mitochondrial localization (MitoCarta2.0) is indicated. See also Figure 4 a-b, Supplementary Figure 4a.

File name: Supplementary Data 4

Description: TIMM44-TurboID proximity labelling, interactome and interactions upon misfolding stress.

TIMM44-TurboID steady state interactome ±doxycycline listed as individual replicates and averaged log2 fold changes. Data presented in Figure 4e. TIMM44-TurboID proximity data set under misfolding stress normalized to the steady state interactome, used for Figure 4f and Supplementary Figure 4d. Pulsed-SILAC translation data of PAM complex components and translocons used in Supplementary Figure 4e listed. SF = Supplementary Figure, dox = doxycycline. Statistical significance was tested by two-sided unpaired t-test without adjustments for multiple testing.
