# Supplementary material for: Protein import motor complex reacts to mitochondrial misfolding by reducing protein import and activating mitophagy
Source: Nat Commun. 2022 Sep 2;13:5164. doi: 10.1038/s41467-022-32564-x (PMC9440083; doi:10.1038/s41467-022-32564-x)
Supplement: Supplementary file 7 — Reporting Summary [file 41467_2022_32564_MOESM7_ESM.pdf]

nature research

Corresponding author(s): Christian Münch

Last updated by author(s): 05/29/2022

Reporting Summary

Nature Research wishes to improve the reproducibility of the work that we publish. This form provides structure for consistency and transparency in reporting. For further information on Nature Research policies, see our Editorial Policies and the Editorial Policy Checklist.

Statistics

For all statistical analyses, confirm that the following items are present in the figure legend, table legend, main text, or Methods section.

n/a	Confirmed	
☐	☒	The exact sample size (*n*) for each experimental group/condition, given as a discrete number and unit of measurement
☐	☒	A statement on whether measurements were taken from distinct samples or whether the same sample was measured repeatedly
☐	☒	The statistical test(s) used AND whether they are one- or two-sided<br>*Only common tests should be described solely by name; describe more complex techniques in the Methods section.*
☒	☐	A description of all covariates tested
☐	☒	A description of any assumptions or corrections, such as tests of normality and adjustment for multiple comparisons
☐	☒	A full description of the statistical parameters including central tendency (e.g. means) or other basic estimates (e.g. regression coefficient) AND variation (e.g. standard deviation) or associated estimates of uncertainty (e.g. confidence intervals)
☐	☒	For null hypothesis testing, the test statistic (e.g. *F*, *t*, *r*) with confidence intervals, effect sizes, degrees of freedom and *P* value noted<br>*Give P values as exact values whenever suitable.*
☒	☐	For Bayesian analysis, information on the choice of priors and Markov chain Monte Carlo settings
☒	☐	For hierarchical and complex designs, identification of the appropriate level for tests and full reporting of outcomes
☐	☒	Estimates of effect sizes (e.g. Cohen's *d*, Pearson's *r*), indicating how they were calculated

Our web collection on statistics for biologists contains articles on many of the points above.

Software and code

Policy information about availability of computer code

Data collection	NextSeq Control Software 2.2.0.4, Illumina Experiment Manager; CQ1 Software, Tune 2.9, Xcalibur 4.0, , Li-Cor Odyssey/ Image Studio, FACSDiva 8.0.1
Data analysis	Python 3 with cutadapt 2.8 and Bowtie2 2.3.0, MAGeCK v0.5.6;NGS data analysis, Proteome Discoverer 2.4;MS raw data analysis, ImageJ 1.53c with JACoP plug-in;microscopy and co-localization analysis; Perseus 1.6.5.0.; Clustering, PANTHER 15.0;Reactome analysis, R studio 1.2.5033 ggplot2, stringr, ggridges and tidyverse packages;Data visualization, STRING-DB 11.0;Protein-protein networks, GraphPad Prism 6 and 9 ;Box plots and bar graphs, Flowjo v10; Flow cytometry raw data analysis, Image Studio lite 5.2; immunoblots, Microsoft Excel 2016, Adobe Illustrator CS6

For manuscripts utilizing custom algorithms or software that are central to the research but not yet described in published literature, software must be made available to editors and reviewers. We strongly encourage code deposition in a community repository (e.g. GitHub). See the Nature Research guidelines for submitting code & software for further information.

Data

Policy information about availability of data

All manuscripts must include a data availability statement. This statement should provide the following information, where applicable:

- Accession codes, unique identifiers, or web links for publicly available datasets
- A list of figures that have associated raw data
- A description of any restrictions on data availability

Flow cytometric pseudocolor plots for mt-mKEIMA assays and membrane potential measurements via TMRE, as well as images of all immunoblots used for this study can be found in the Source Data file. Original full size microscopy images are accessible here doi:10.17632/4dc8hyjwhw.1. The analyzed CRISPR-screen data are available in Supplementary Data 1. Next generation sequencing raw files are available at doi:10.17632/s3jtdhjdbs.1. The mass spectrometry proteomics raw

data have been deposited to the ProteomeXchange Consortium via the PRIDE 73 partner repository with the dataset identifier PXD022560 , PXD022524 and PXD022530. The Homo sapiens SwissProt database (TaxID = 9606, v. 2017-10-25) was used for proteome analyses.

Field-specific reporting

Please select the one below that is the best fit for your research. If you are not sure, read the appropriate sections before making your selection.

☒ Life sciences ☐ Behavioural & social sciences ☐ Ecological, evolutionary & environmental sciences

For a reference copy of the document with all sections, see nature.com/documents/nr-reporting-summary-flat.pdf

Life sciences study design

All studies must disclose on these points even when the disclosure is negative.

Sample size	No calculations were performed to predetermine sample size, sample sizes were based on in the field accepted standards (three biological replicates were used with 5000-10000 cells in flow cytometric experiments, 100 cells in microscopic experiments and approximately 800,000-1,000,000 cells (one 6-well) per proteomic or immunoblotting sample).
Data exclusions	C.elegans worms and embryos with clear developmental problems or improperly mounted were excluded from our analysis.<br>Common contaminants (`contaminants.fasta` provided with MaxQuant) were excluded for mass spectrometry experiments from further analysis.
Replication	All attempts of replication were successful. Experiments were carried out in minimum 3 biological replicates as indicated in the results, but for Supplementary Figure 4 l,k.
Randomization	No specific method of randomization was used as control and treatment groups were derived from the same cell lines.
Blinding	No blinding conditions were used as experiments and data analysis were performed by the same person and knowledge of the different conditions was required for sample preparation.

Reporting for specific materials, systems and methods

We require information from authors about some types of materials, experimental systems and methods used in many studies. Here, indicate whether each material, system or method listed is relevant to your study. If you are not sure if a list item applies to your research, read the appropriate section before selecting a response.

Materials & experimental systems

n/a	Involved in the study
☐	☒ Antibodies
☐	☒ Eukaryotic cell lines
☒	☐ Palaeontology and archaeology
☐	☒ Animals and other organisms
☒	☐ Human research participants
☒	☐ Clinical data
☒	☐ Dual use research of concern

Methods

n/a	Involved in the study
☒	☐ ChIP-seq
☐	☒ Flow cytometry
☒	☐ MRI-based neuroimaging

Antibodies

Antibodies used	anti-beta-Actin (SantaCruz, sc69879, 1:5000)<br>anti-GrpEL1 (Proteintech, 12720-1-AP, 1:1000)<br>anti-HSP60 (Abcam, ab4679, 1:10,000)<br>anti-HSPA9 (Abcam, JG1 clone, ab2799, 1:2000)<br>anti-LONP1 (Proteintech,15440-1-AP, 1:1000)<br>anti-PAM16 (Proteintech, 15321-1-AP, 1:1000)<br>anti-phospho (S65)-Ubiquitin (Boston Biochem, A110, 1:1000)<br>anti-PINK1 (CST, D8G3 clone, 6946, 1:1000)<br>anti-TIMM23 (Proteintech, 11123-1-AP, 1:1000)<br>anti-TIMM44 (Proteintech, 13859-1-AP, 1:1000)<br>anti-TOMM20 (SantaCruz, sc17764, 1:1000)<br>anti-TOMM40 (SantaCruz, sc 365467, 1:1000)<br>anti-mouse-IgG-680RD (Li-Cor 926-68072, 1:10,000)<br>anti-mouse-IgG-800CW (Li-Cor 926-32210, 1:15,000)<br>anti-rabbit-IgG-680 (Li-Cor 926-68073, 1:15,000)<br>anti-rabbit-IgG-800CW (Li-Cor 926-32213, 1:10,000)

Validation	anti-β-Actin validated by SantaCruz via overexpression experiments (https://www.scbt.com/p/beta-actin-antibody-ac-15).<br>anti-GrpEL1 validated by siRNA doi: 10.1038/s41467-020-20597-z.<br>anti-HSP60 validated by knockdown doi.org: 10.1186/s13046-021-02049-8<br>anti-HSPA9 and antiLONP1 were validated in this study by knockdown experiments Supplementary Figure 2a, b.<br>anti-PAM16 was validated knockdown experiments in Supplementary Figure 2f.<br>anti-phospho (S65)-Ubiquitin was validated by immunoblot detection of recombinant phospho-ubiquitin chains by R&D systems.<br>anti-PINK1 was validated by Cell Signaling Technology using cDNA overexpression.<br>anti-TIMM23 was validated in doi: 10.7554/eLife.65158 by knockdown.<br>anti-TIMM44 validated by siRNA doi: 10.1038/s41467-020-20597-z.<br>anti-TOMM20 and anti-TOMM40 were checked for detection of protein at the expected size in whole cell lysates and for mitochondrial localization via fluorescence microscopy by SantaCruz.

Eukaryotic cell lines

Policy information about cell lines

Cell line source(s)	HeLa Flp-In TRex (Le Guerroué et al 2017,https://doi.org/10.1016/j.molcel.2017.10.029), HeLa (ATCC), Hek 293T (ATCC)
Authentication	Cell lines were not further authenticated.
Mycoplasma contamination	The cell lines were PCR-tested and confirmed negative for mycoplasma.
Commonly misidentified lines (See ICLAC register)	No commonly misidentified cell lines were used.

Animals and other organisms

Policy information about studies involving animals; ARRIVE guidelines recommended for reporting animal research

Laboratory animals	C.elegans with following strain was used in this study: N2, CHP124 (mCherry::lgg-1 (syb297) x unc-119(ed3) III; ddIs105[sir-2.2::TY1::EGFP::3xFLAG(92C12) + unc-119(+)].) All animals were egg-laying young adults hermaphrodites shifted from a diet of E.coli OP50 or HT115
Wild animals	The study did not involve wild animals.
Field-collected samples	The study did not involve field-collected samples
Ethics oversight	No ethical approval required for C.elegans experiments.

Note that full information on the approval of the study protocol must also be provided in the manuscript.

Flow Cytometry

Plots

Confirm that:

☒ The axis labels state the marker and fluorochrome used (e.g. CD4-FITC).

☒ The axis scales are clearly visible. Include numbers along axes only for bottom left plot of group (a 'group' is an analysis of identical markers).

☒ All plots are contour plots with outliers or pseudocolor plots.

☒ A numerical value for number of cells or percentage (with statistics) is provided.

Methodology

Sample preparation	Adherend stable cell line cells were detached by digestion with 0.25% trypsin/EDTA, resuspended in RPMI 10% FBS, sedimented by centrifugation and resuspended in PBS. Samples were hold on ice and dark until measurement.
Instrument	LSRII/Fortessa flow cytometer, FACSAria III cell sorter, FACSymphony™ A5 Cell Analyzer (BD Biosciences, Heidelberg, Germany)
Software	BD FACS Diva (BD Biosciences) for acquisition and FlowJo software V10 (Treestar) for analysis
Cell population abundance	HeLa FlpIn TRex Parkin mt-mKEIMA were about 96% mKEIMA-positive and was sorted for mKEIMA, HeLa FlpIn TRex Parkin mt-mKEIMA with PINK1 KO was about 3% mKEIMA-positive. The mKEIMA-positive gate was set that maximum 0.1% of empty Hela FlpIn TRex cells were present.<br>TMRE measurements were done with PE-A (TMRE)-positive cells and compared to unstained and CCCP-treated cells from the same day.

For FACS data quantification, pseudocolor or histogram plots are provided in the supplements or Source Figures 1 and 2 (doi:10.17632/rrbk8b4jpz.1 (when published) https://data.mendeley.com/datasets/rrbk8b4jpz/draft?a=ffbc18cb-2b6e-4a83-b14b-b966e5bda5ab (preview)).

Gating strategy

Total event population was gated in FSC-A/SSC-A excluding debris, gated for single cells by FCS-A/H,SSC-A/H and fluorescent cells either TMRE or mt-mKEIMA pH4 (basal level)-positive were used for further analysis.

☒ Tick this box to confirm that a figure exemplifying the gating strategy is provided in the Supplementary Information.
