# Supplementary material for: Protein import motor complex reacts to mitochondrial misfolding by reducing protein import and activating mitophagy
Source: Nat Commun. 2022 Sep 2;13:5164. doi: 10.1038/s41467-022-32564-x (PMC9440083; doi:10.1038/s41467-022-32564-x)

Source data description

Source Data page 1-2: Pseudo-color dot plots of mt-mKEIMA experiments which are the bases for Figure 2c, g, 3b and f statistical analyses, are shown as pseudo colors plot for at least 5,000 cells per replicate.

Source Data page 3-4: Histograms showing TMRE results for individual replicates, which are the bases for Figure 2d, h and 3c, f statistical analyses, are shown as histograms for at least 5,000 cells per replicate.

Source Data page 5-10: Immunoblots full images and all replicates used for quantification.

Source Data Microscopy: Original fluorescence microscopy images for MTS-EGFP with Mitotracker Deep Red FM used for Figure 2a, b, and mt-mKEIMA (405 nm, mitochondrial and 561 nm, lysosomal mt-mKEIMA) live cell microscopy. Description of channels and image nomenclature included in zip-file. Data is accessible via Mendeley data storage: doi:10.17632/4dc8hyjwhw.1

Source Data,mt-mKEIMA flow cytometry, page 1

Fig. 2c, 3g

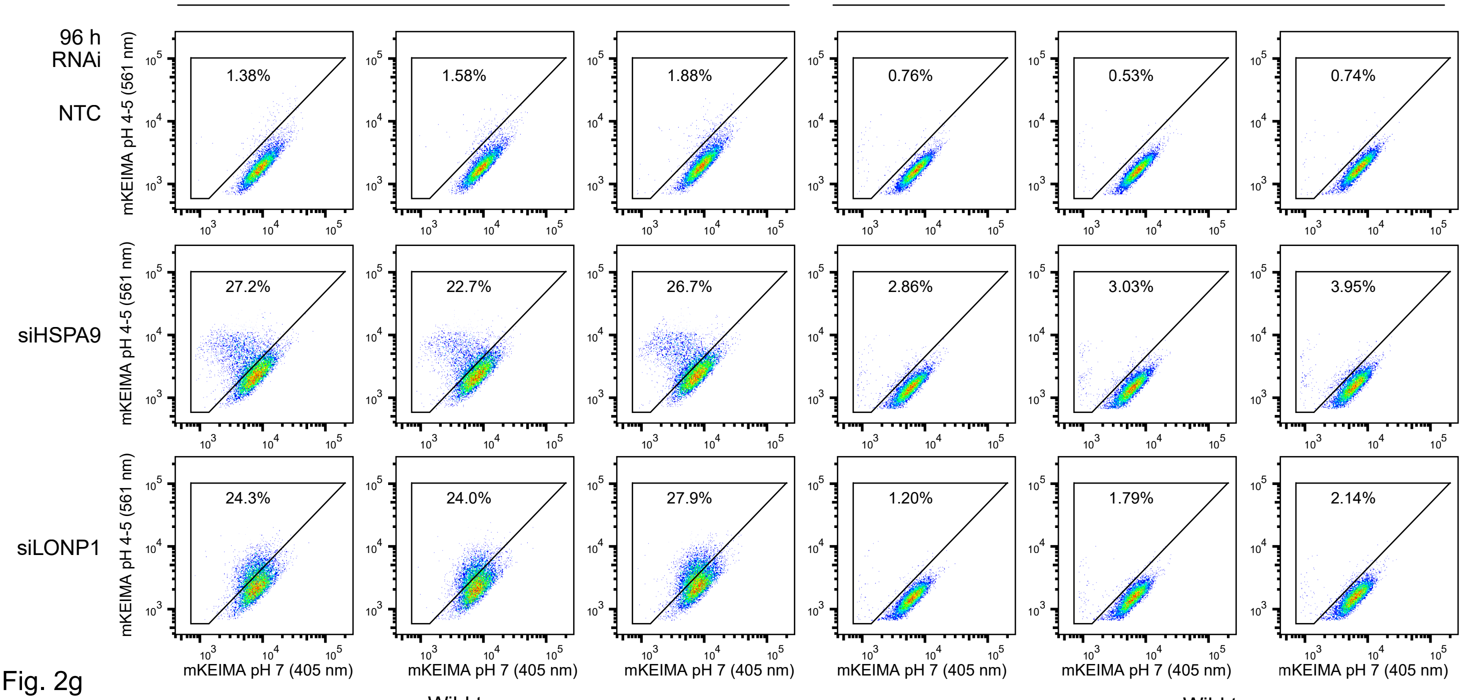

Fig. 2g

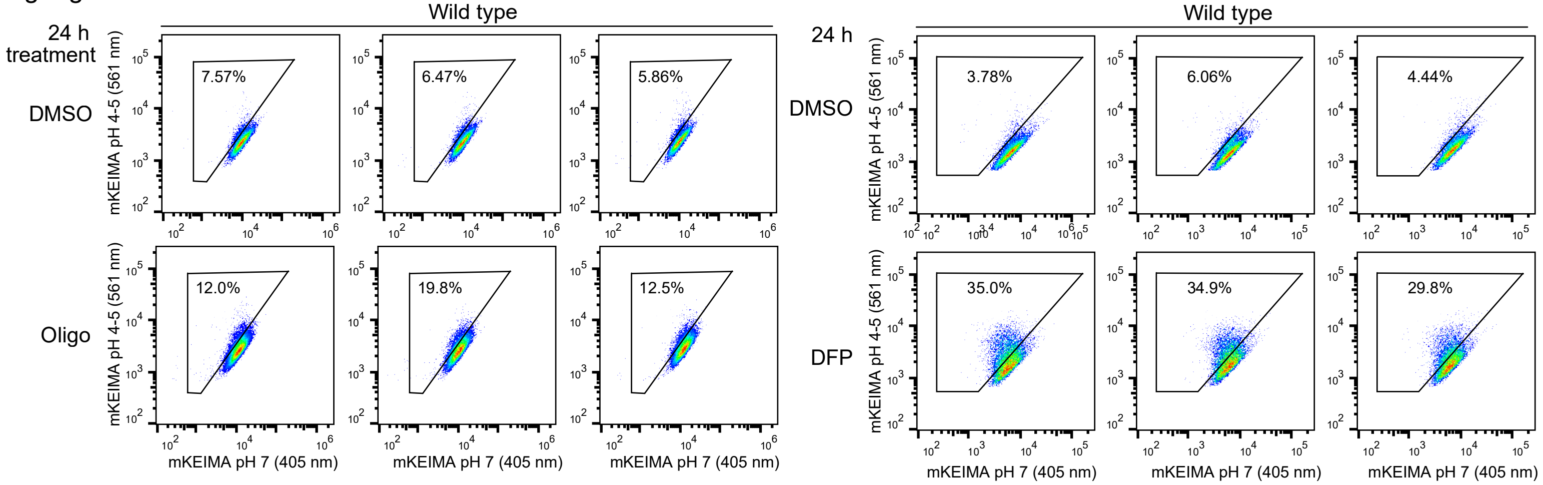

Fig. 3b

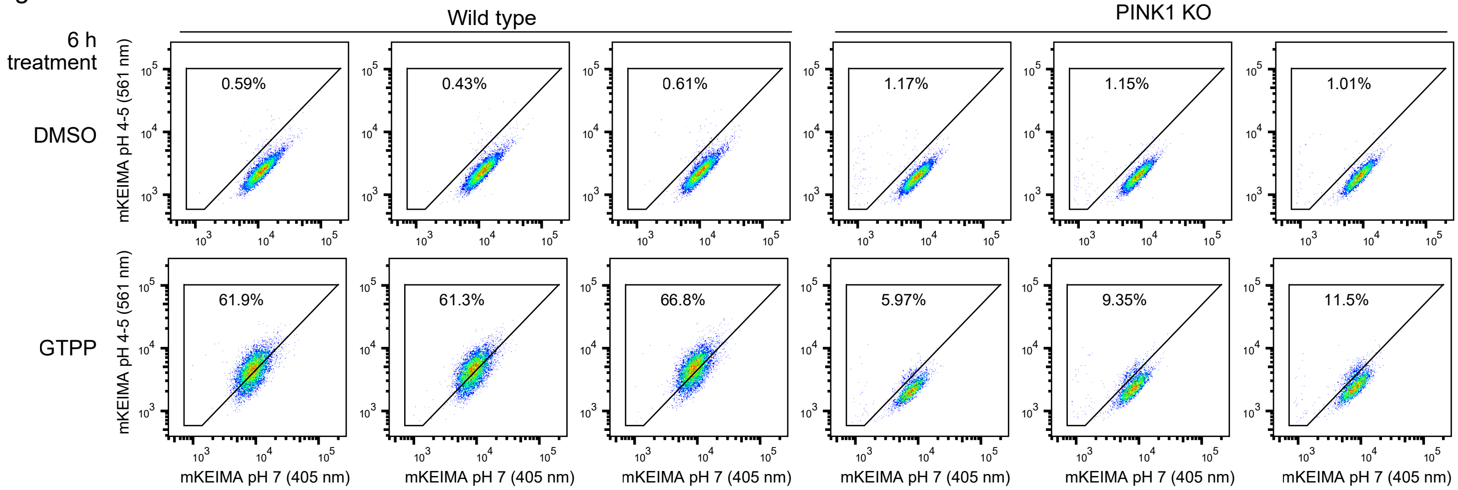

Source Data, mt-mKEIMA flow cytometry, page 2

Supplementary Figure 2e

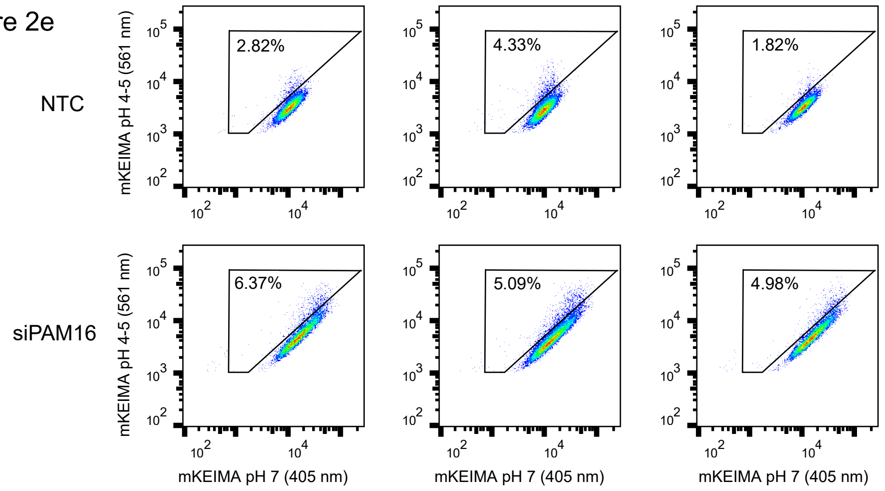

Supplementary Figure 4g

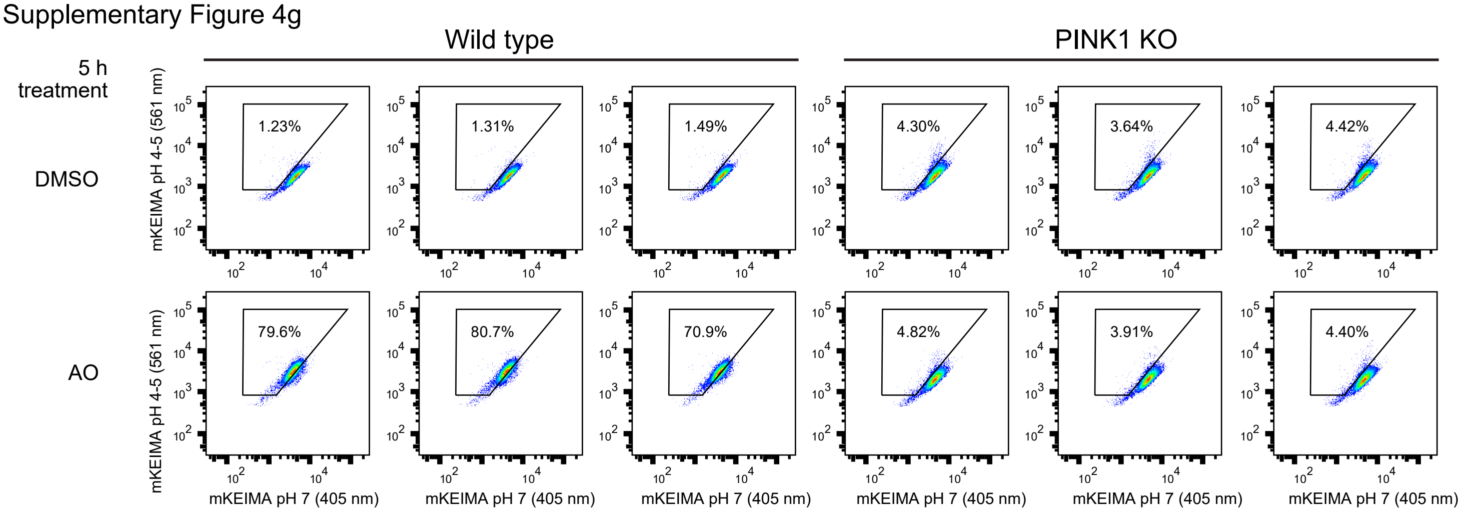

Source Data TMRE-positive cells, page 3

Fig. 2d

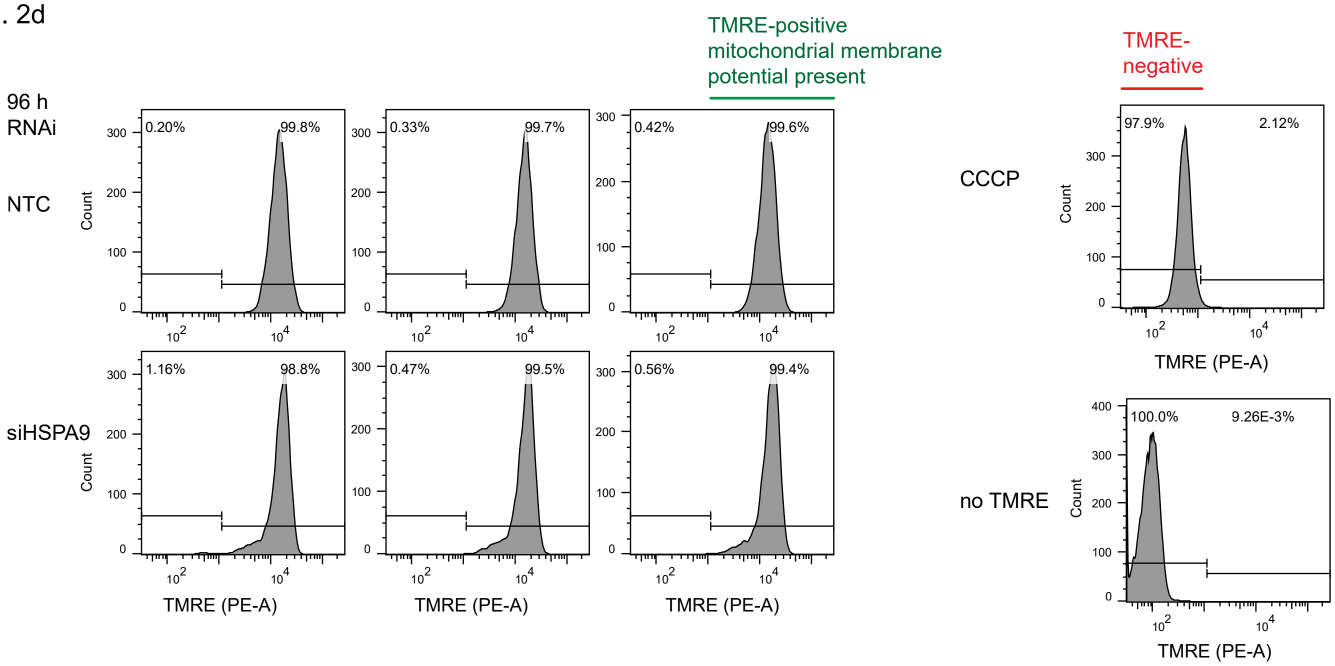

Fig. 2h

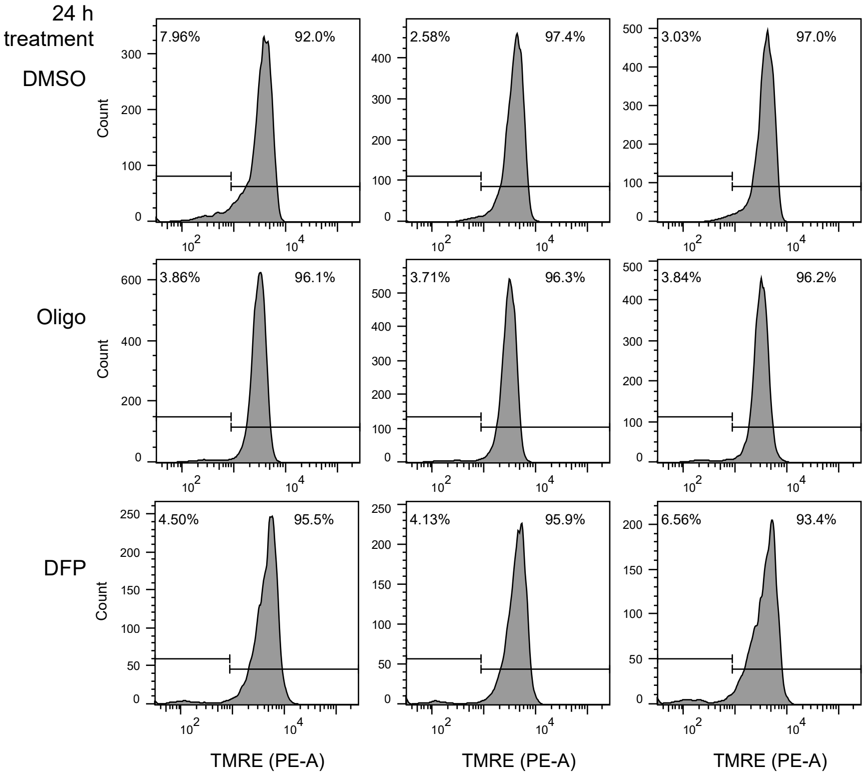

Source Data, TMRE-positive cells, page 4

Fig. 3c

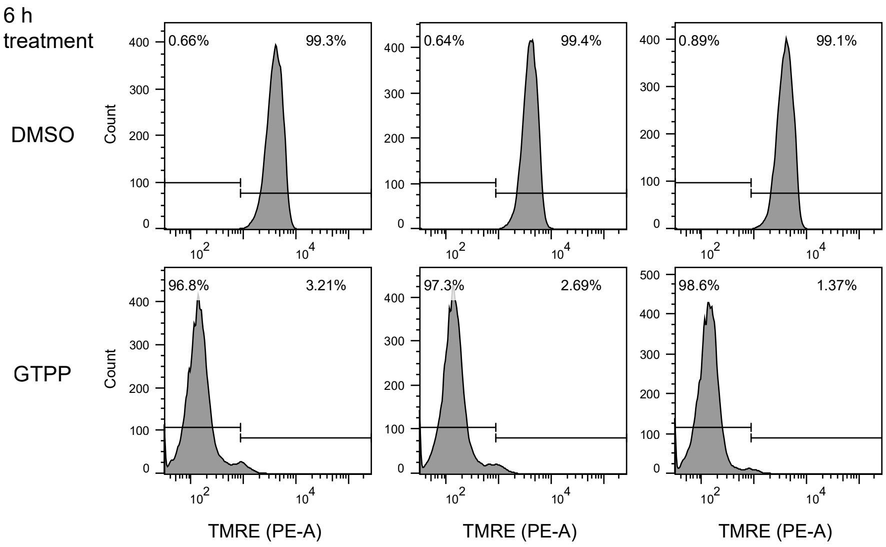

Fig. 3f

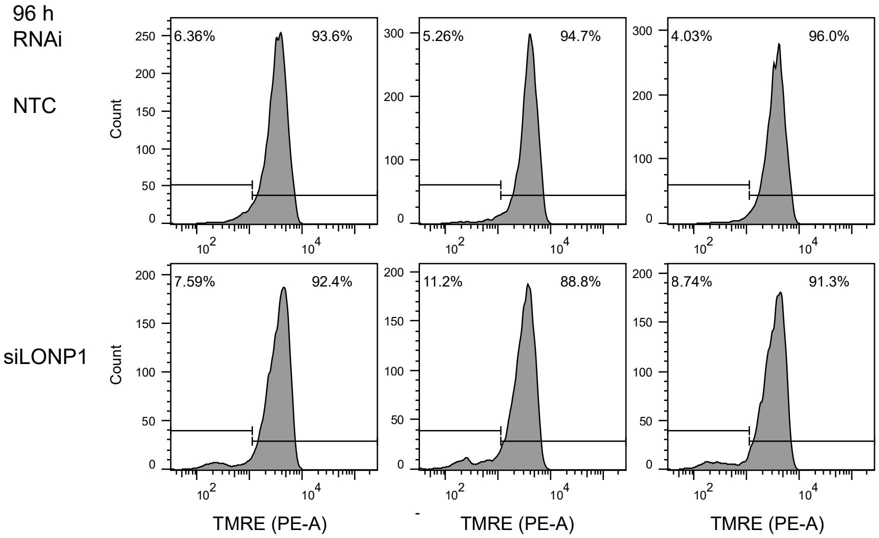

Fig. 4c

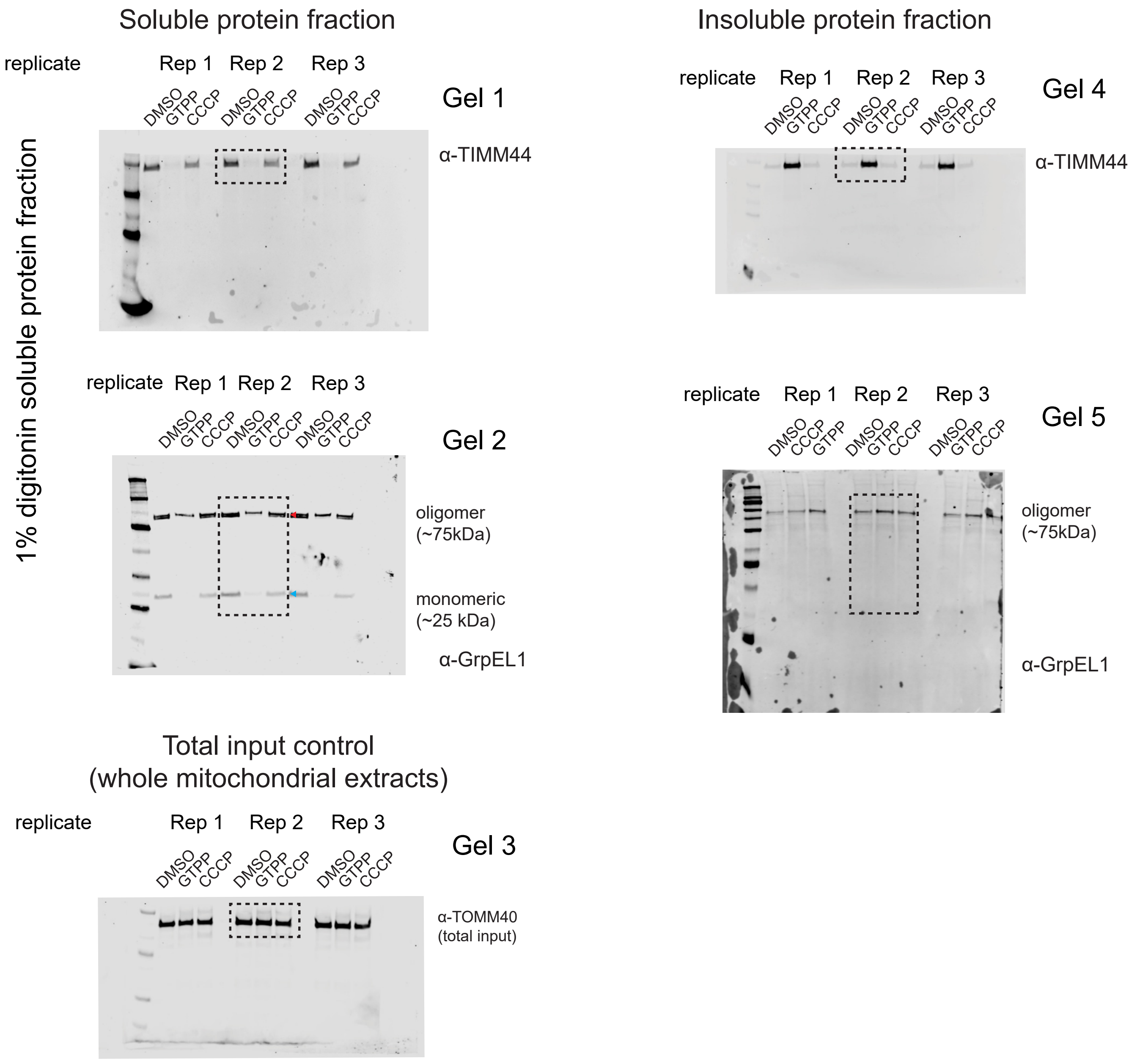
Soluble protein fraction
replicate
Rep 1
Rep 2
Rep 3
DMSO GTPP CCCP DMSO GTPP CCCP DMSO GTPP CCCP
Gel 1
α-TIMM44
1% digitonin soluble protein fraction
replicate
Rep 1
Rep 2
Rep 3
DMSO GTPP CCCP DMSO GTPP CCCP DMSO GTPP CCCP
Gel 2
oligomer (~75kDa)
monomeric (~25 kDa)
α-GrpEL1
Insoluble protein fraction
replicate
Rep 1
Rep 2
Rep 3
DMSO GTPP CCCP DMSO GTPP CCCP DMSO GTPP CCCP
Gel 4
α-TIMM44
replicate
Rep 1
Rep 2
Rep 3
DMSO CCCP GTPP DMSO GTPP CCCP DMSO GTPP CCCP
Gel 5
oligomer (~75kDa)
α-GrpEL1
Total input control (whole mitochondrial extracts)
replicate
Rep 1
Rep 2
Rep 3
DMSO GTPP CCCP DMSO GTPP CCCP DMSO GTPP CCCP
Gel 3
α-TOMM40 (total input)

Source Data, immunoblots, page 6

Supplementary Fig. 2a, b

Replicate 1

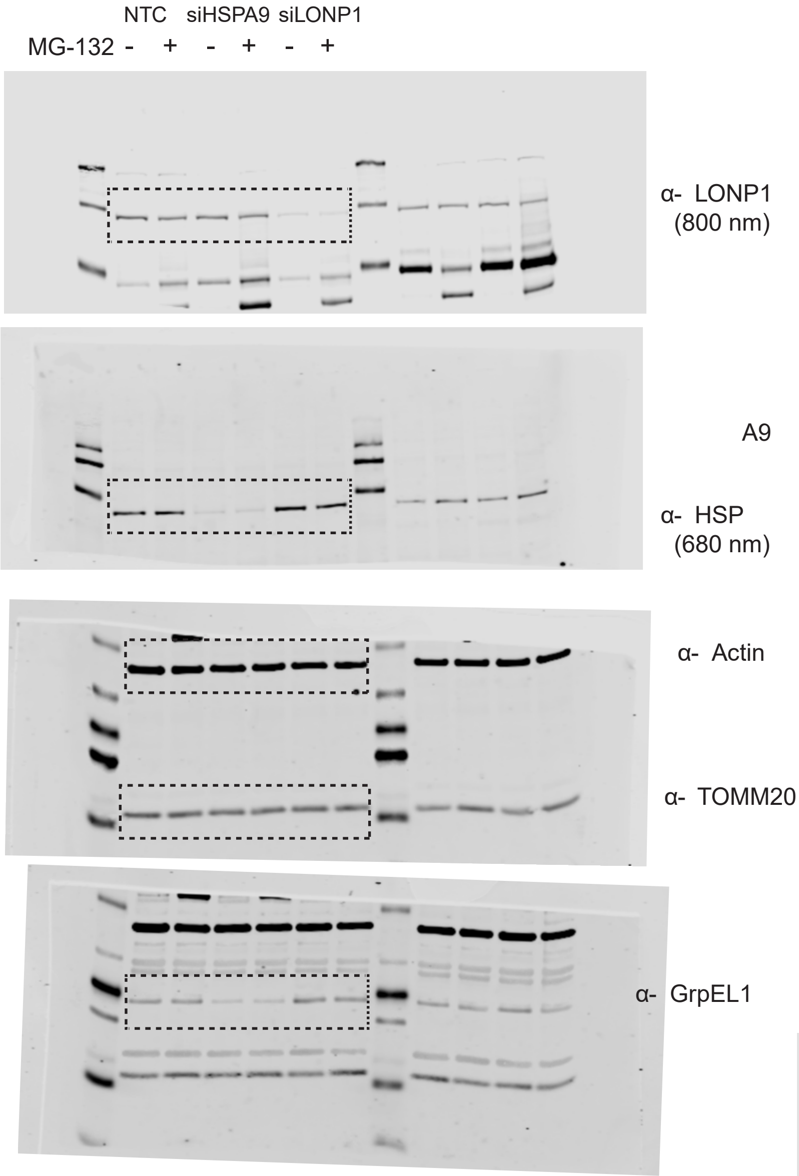

Replicate 2 (Supplementary Fig. 2b)

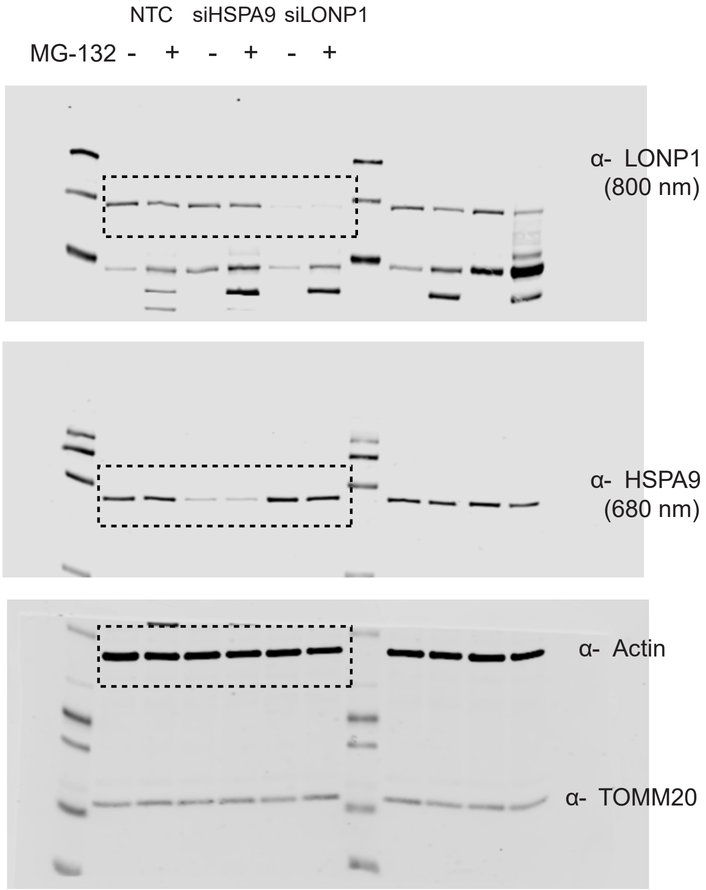

Replicate 3 (Supplementary Fig. 2b)

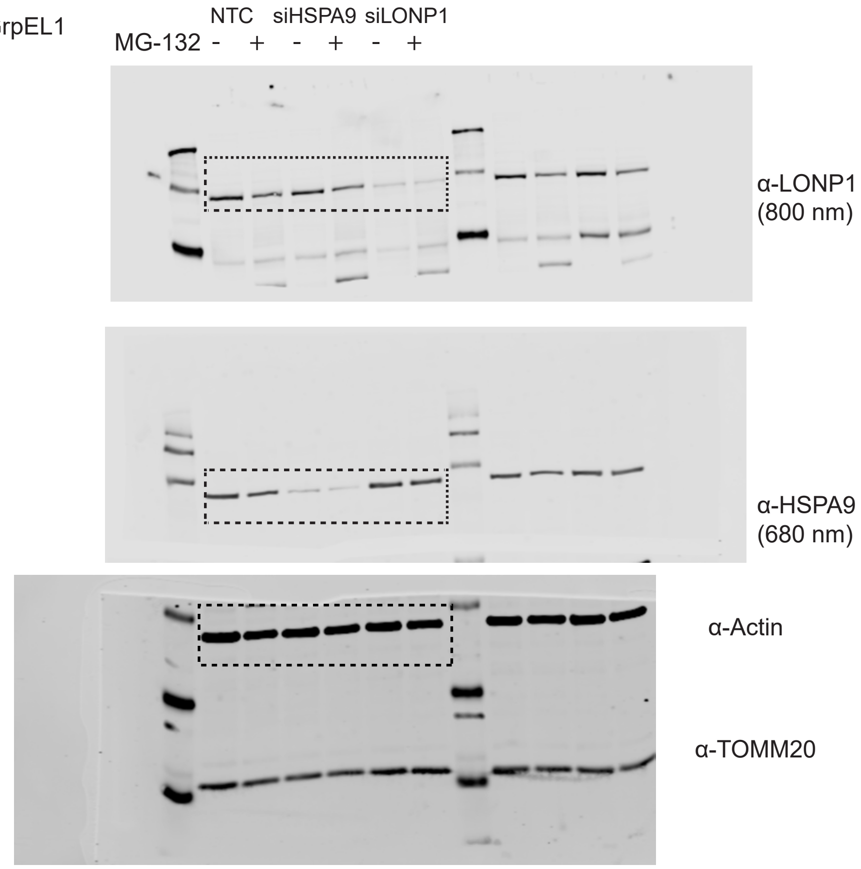

Source Data, immunoblots, page 7

Supplementary Fig. 2c

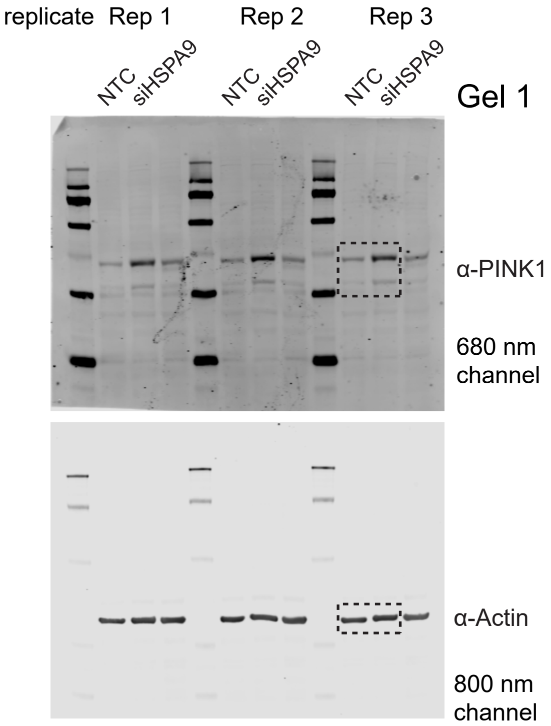

Supplementary Fig. 2f

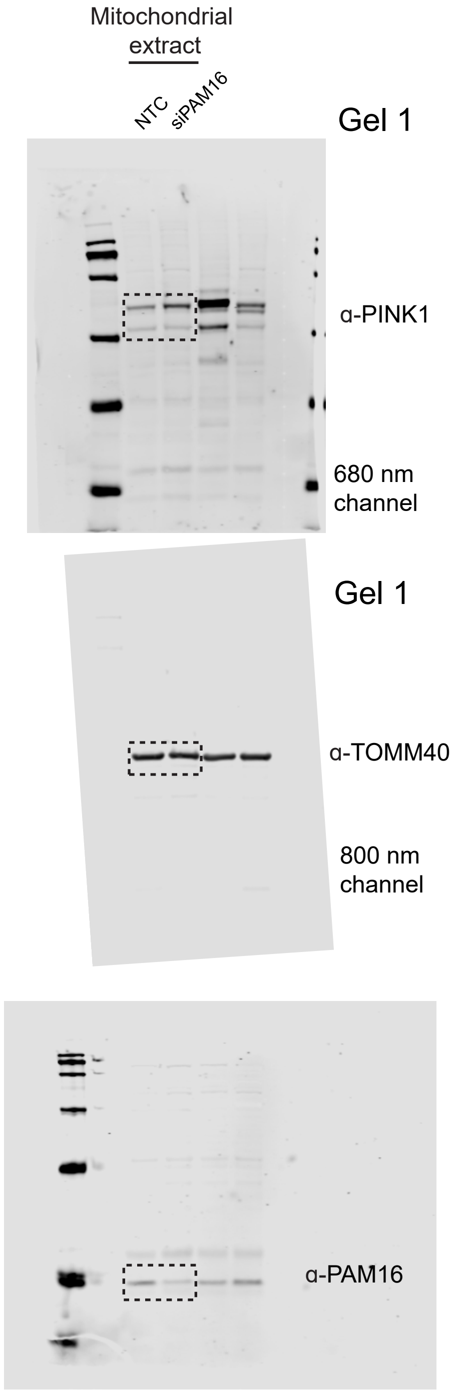

Source Data, immunoblots, page 8

Supplementary Fig. 3d

Mitochondrial extract

DMSO GTPP CCCP

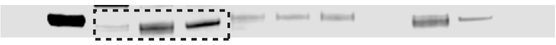

α-PINK1

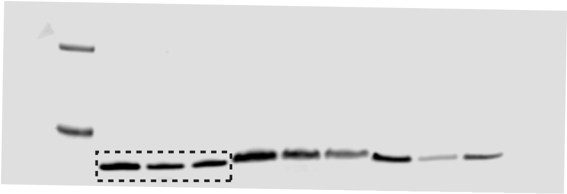

α-TIMM23

replicate Rep 1 Rep 2 Rep 3

DMSO GTPP CCCP DMSO GTPP CCCP DMSO GTPP CCCP

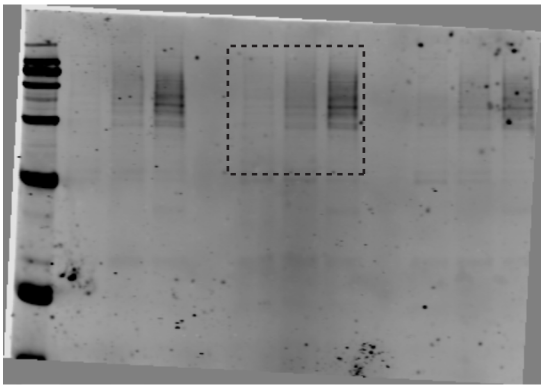

α-phospho-S65-Ub

680 nm channel

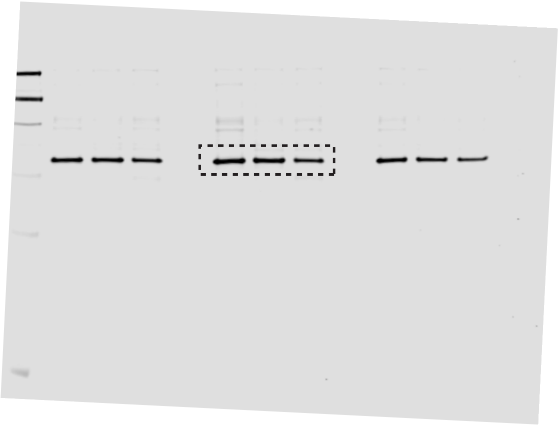

α-TOMM40

800 nm channel

Mitochondrial extracts (Total input as in Fig. 4c)

replicate Rep 1 Rep 2 Rep 3

DMSO GTPP CCCP DMSO GTPP CCCP DMSO GTPP CCCP

Gel 3
loading control
see page 5
anti-TOMM40

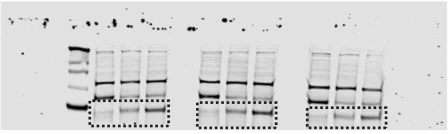

α-PINK1

800 nm channel

Source Data, immunoblots, page 9

Supplementary Fig. 3i

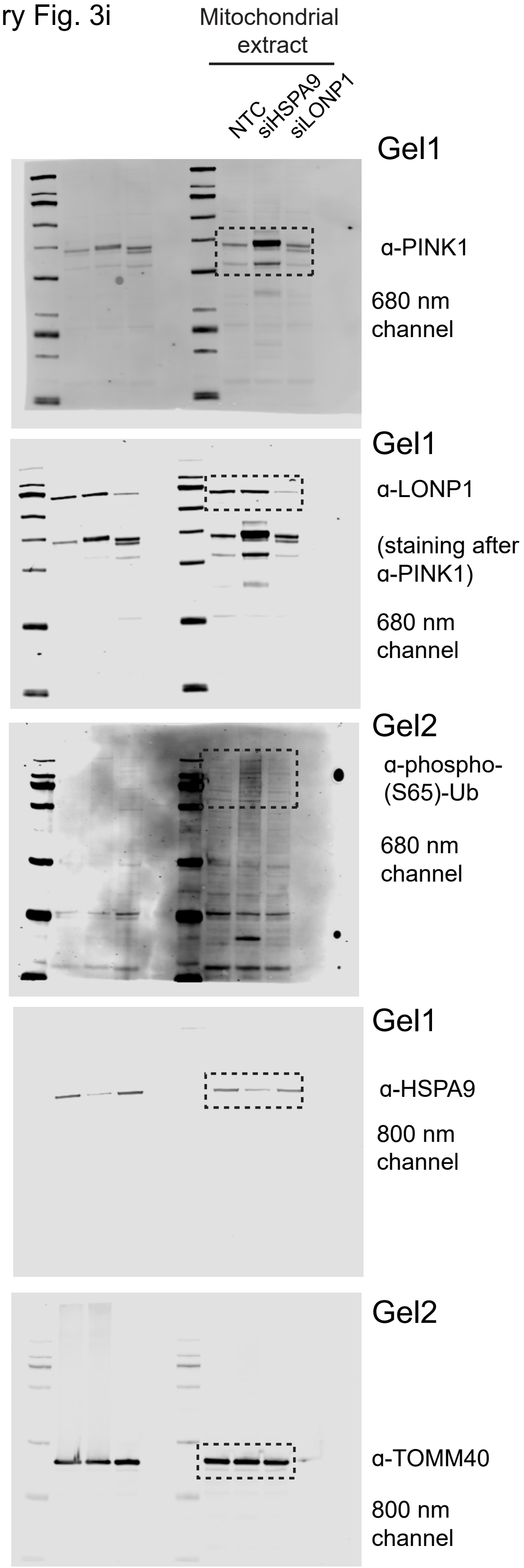

Supplementary Fig. 3j

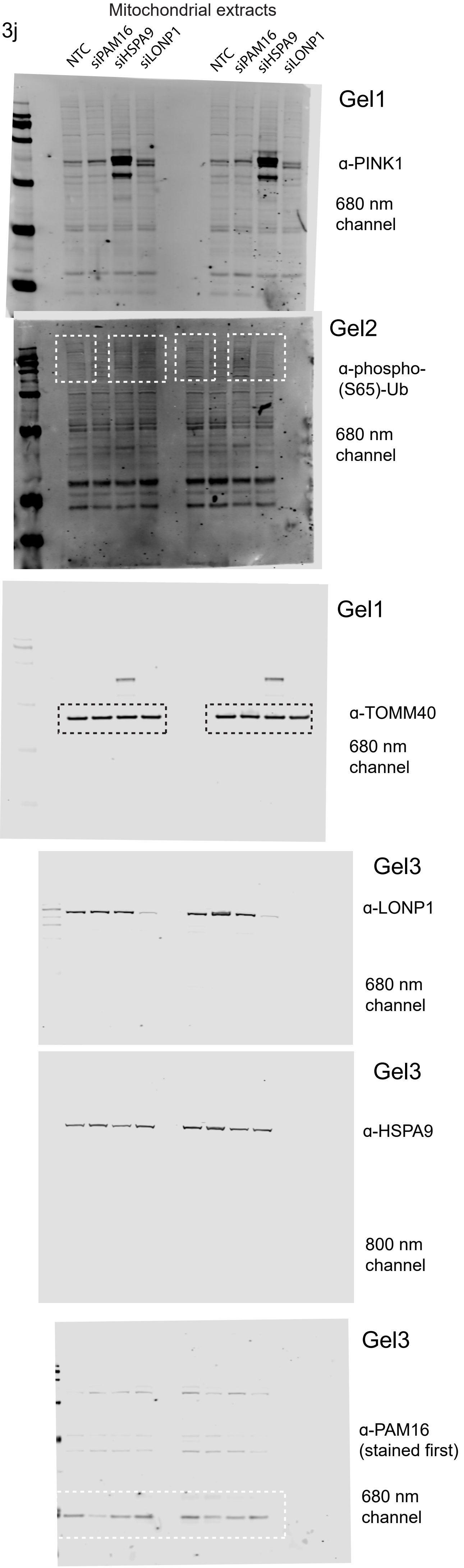

Source Data, immunoblots, page 10

Supplementary Fig. 3k

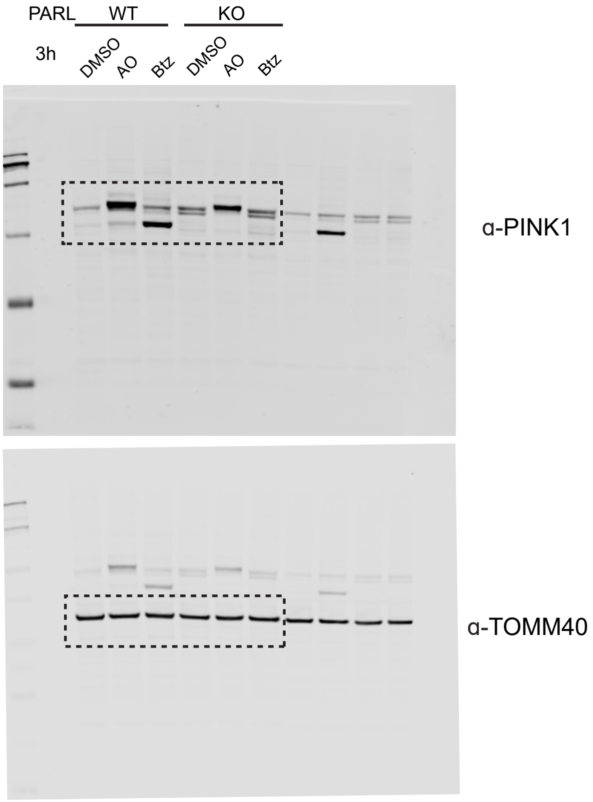

Supplementary Fig. 3l

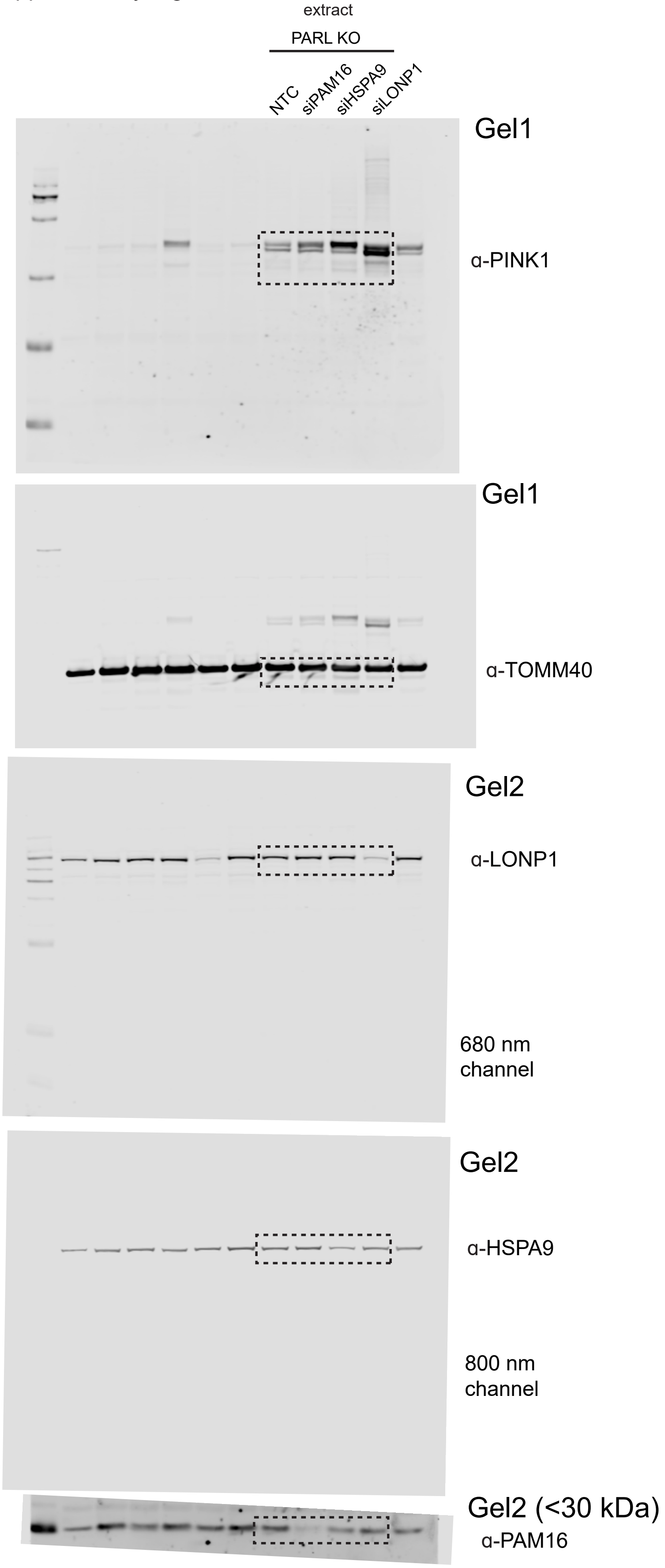

Supplement: Supplementary file 8 — Source Data [file 41467_2022_32564_MOESM8_ESM.pdf]
